# Genome-wide association study, population structure, and genetic diversity of the tea plant in Guizhou Plateau

**DOI:** 10.1186/s12870-024-04761-x

**Published:** 2024-01-30

**Authors:** Yihan Wang, Suzhen Niu, Xinyue Deng, Dingchen Bai, Zhengwu Chen, Xiuling Deng, Dejun Huang

**Affiliations:** 1 College of Tea Science, Guizhou University, Guiyang, Guizhou Province 550025 China; 2 The Key Laboratory of Plant Resources Conservation and Germplasm Innovation in Mountainous Region (Ministry of Education), Institute of Agro-Bioengineering, Guizhou University, Guiyang, Guizhou Province 550025 China; 3 School of Architecture, Guizhou university, Guiyang, Guizhou Province 550025 China; 4 lnstitute of Tea, Guizhou Academy of Agricultural Sciences, Guiyang, Guizhou Province 550006 China

**Keywords:** Genotyping-by-sequencing, Tea plant, Population structure, Genetic diversity, Genome-wide association study, Guizhou plateau

## Abstract

**Background:**

Guizhou Plateau, as one of the original centers of tea plant, has a profound multi-ethnic cultural heritage and abundant tea germplasm resources. However, the impact of indigenous community factors on the genetic diversity, population structure and geographical distribution of tea plant is still unclear.

**Results:**

Using the genotyping-by-sequencing (GBS) approach, we collected 415 tea plant accessions from the study sites, estimated genetic diversity, developed a core collection, and conducted a genome-wide association study (GWAS) based on 99,363 high-quality single-nucleotide polymorphisms (SNPs). A total of 415 tea accessions were clustered into six populations (GP01, GP02, GP03, GP04, GP05 and GP06), and the results showed that GP04 and GP05 had the highest and lowest genetic diversity (*Pi* = 0.214 and *Pi* = 0.145, respectively). Moreover, 136 tea accessions (33%) were selected to construct the core set that can represent the genetic diversity of the whole collection. By analyzing seven significant SNP markers associated with the traits such as the germination period of one bud and two leaves (OTL) and the germination period of one bud and three leaves (OtL), four candidate genes possibly related to OTL and OtL were identified.

**Conclusions:**

This study revealed the impact of indigenous communities on the population structure of 415 tea accessions, indicating the importance of cultural practices for protection and utilization of tea plant genetic resources. Four potential candidate genes associated with the OTL and OtL of tea plant were also identified, which will facilitate genetic research, germplasm conservation, and breeding.

**Supplementary Information:**

The online version contains supplementary material available at 10.1186/s12870-024-04761-x.

## Background

Biodiversity is the ecological complex formed by organisms, environmental factors and various related ecological processes [1, 2]. It is explored at three levels or types, including genetic diversity, species diversity and ecosystem diversity [3]. Biodiversity revitalizes the earth by supporting and sustaining healthy ecosystems, and lays the foundation for human survival and development [4, 5]. However, over-exploitation of resources, social transformation, climate change and pollution are posing serious threats to biodiversity [6–8]. Therefore, it is necessary to effectively protect biodiversity to maintain the ecosystem processes and ultimately sustain human life [9, 10].

The traditional culture of indigenous people and ecological knowledge of smallholder farmers contribute to biodiversity conservation and inhibit the rapid loss of biodiversity around the world [11, 12]. Previous studies have demonstrated that traditional management practices, customs and economic exchanges, including religious or ancestral worship ceremonies [13], dietary practices [14, 15], seed exchange systems [16] and marriage rituals [17] play positive roles in biodiversity conservation. Moreover, these practices and customs have also been related to preserving landraces and old trees with medicinal, nutritional and aesthetic values [18–20].

Tea (*Camellia sinensis* (L.) O. Kuntze), as one of the three most popular non-alcoholic beverages in the world, contains numerous secondary metabolites such as caffeine, amino acids, phenolics, terpenes and volatiles, which contribute to economic values, pleasant flavor and health benefits of tea [21]. It is known that the history of tea drinking in China can be traced back to the beginning of the 5th century AD until the end of the 6th century AD, and Chinese people consume tea for different medicinal and edible purposes and also as mixed or pure drinks [22]. Tea plant is an economic crop with tender buds and leaves in the lateral branches as its product organs [23]. The germination period of one bud and two leaves (OTL) and the germination period of one bud and three leaves (OtL) are the major agronomic traits of tea plant. Due to the relatively low temperature in early spring, early OTL and OtL tea plants can avoid a large amount of pests and diseases during the first round of harvesting, accumulating numerous natural compounds that can improve the quality of tea [23]. Early spring tea has higher economic value, therefore, breeding early OTL and OtL varieties is a valuable goal [24].

Tea plants are believed to be originated in southwestern China, which includes Guizhou Plateau and the adjacent provinces [25]. The Guizhou Plateau is a multi-ethnic area and home to six groups of indigenous people such as Miao (Miao Man, MM) and Gelao (Pu Ren, PR), who have the longest history of living in Guizhou Plateau, as well as Tujia and Yi (Di Qiang, DQ), Buyi (Bai Yue, BY) and Han (Han Ren, HR) [26]. Guizhou Plateau has a superior geographical location, profound cultural heritage and rich biodiversity. This region has preserved abundant tea germplasm resources, with wild type accessions, ancient landraces and modern landraces [27]. However, with the degradation of habitat, the reduction of genetic diversity, and the trend of monoculture farming [28, 29], it is increasingly important to understand the current population structure and genetic diversity of tea plant in the Guizhou Plateau. Although many studies have confirmed the importance of traditional agricultural management knowledge for biodiversity conservation [30], the complex interaction between the customs and the migration of indigenous people and the population structure of tea accessions has not been studied.

In this study, we integrated ethnobotanical and molecular genetic methods to explore the impact of migration, customs and economic exchanges of indigenous communities in the Guizhou Plateau on the population structure, geographical distribution of tea plant, and the protection of genetic diversity. We collected 415 tea germplasm resources from 33 regions in Guizhou Plateau, as well as Zhejiang, Hunan and Fujian Provinces. Based on the genotyping-by-sequencing (GBS) approach, the genetic diversity, population structure and linkage disequilibrium (LD) of 415 tea accessions were studied using large numbers of single-nucleotide polymorphisms (SNPs). Finally, we constructed a core collection of all tea accessions and conducted a genome-wide association study (GWAS) to identify candidate genes that control OTL and OtL of tea plant.

## Results

### Sequencing and variant discovery

**Fig. 1:**
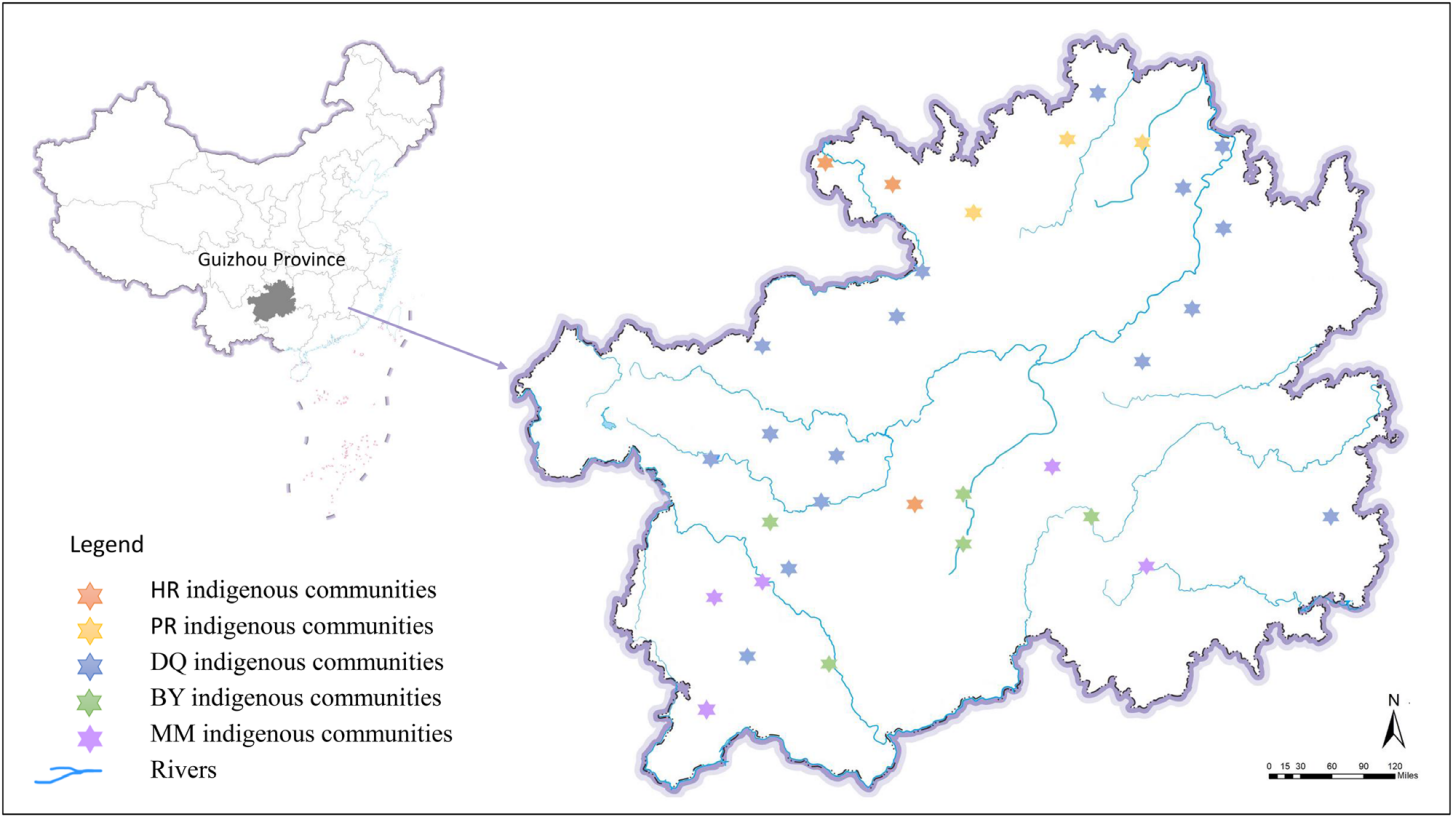
Geographic distribution of the sampling locations. The different colored hexagonal stars represent the geographic distribution of sampling locations of five indigenous communities in Guizhou Plateau [26], including Han (HR) indigenous communities, Gelao (PR) indigenous communities, Tujia and Yi (DQ) indigenous communities, Buyi (BY) indigenous communities and Miao (MM) indigenous communities. The geographic data information used in the map came from field investigations and geographic information surveyed by the Natural Resources Department of Guizhou Province (https://zrzy.guizhou.gov.cn/wzgb/zwgk/zdlyxxgk/dlxxgl/), and the map was drawn using ArcGIS software

A collection of 415 tea plants, including 159 wild type accessions, 174 ancient landraces and 82 modern landraces were used for this study (Additional file 1: Table S1). The geographical distribution of 410 accessions in the indigenous communities of Guizhou Plateau was shown in Fig. 1. The other five accessions were introduced from other provinces and cultivated in the tea garden of Guizhou Plateau. A GBS analysis was performed on all 415 tea accessions using Illumina HiSeq X Ten platform. After filtering low-quality reads, a total of 390.30 Gb clean data were retained, with an average of 0.94 Gb per accession (Additional file 2: Table S1). We mapped the clean reads to a tea reference genome sequence [31], a total of 29,393,327 initial SNPs were obtained from the 415 tea accessions using GATK (v. 3.7.0) software, and 99,363 high-quality SNPs were obtained after filtering, which ensured the reliability and accuracy of subsequent population structure and genetic diversity analyses. The average heterozygosity rate and the average missing rate for per accession were 6.98% and 13.81%, respectively (Additional file 2: Table S2). The distribution of SNPs across chromosomes reveals the genomic landscape of SNPs in the tea population. The SNPs were unevenly distributed on 15 chromosomes, and the lowest and highest SNP densities were detected on chromosomes 5 and 1, respectively (Additional file 5: Fig. S4). At the genomic level, we found that more than half of the SNPs (78.05%) had base transitions, and the transition vs. transversion (ts/tv) ratio was 3.56. The C/G transversion and the C/T transition occurred at the lowest and highest frequencies, respectively. The frequencies of the C/G, G/T, A/T and A/C transversions were 4.10%, 5.60%, 6.79% and 5.46%, respectively, and the frequencies of two-type transitions were similar (39.39% for C/T and 38.66% for A/G) (Additional file 2: Table S3).

### Genetic diversity analysis

The nucleotide diversity (*Pi*), minor allele frequency (*MAF*), observed heterozygosity (*Ho*) and inbreeding coefficient (*Fis*) are used as indicators of genetic diversity, which can evaluate the genetic structure and diversity of the population [32, 33]. *Pi*, *MAF*, *Ho* and *Fis* of 415 accessions were 0.226, 0.144, 0.073 and 0.691, respectively (Table 1). Further analysis of the genetic diversity of tea plant populations from three cultivation status showed that *Pi*, *Ho*, *MAF*and *Fis* of the wild type accessions (WA) population were significantly higher than those of ancient landraces (AL) population and modern landraces (ML) population. *Pi *and *MAF* for the tea plant population in AL were significantly higher than those for the tea plant population in ML. We compared the genetic diversity of tea plant populations from MM, PR, BY, DQ and HR indigenous communities. *Pi*, *Ho* and *MAF* of the tea plant populations from MM and PR exhibited significantly higher compared with those of tea plant populations from BY, DQ and HR. *Pi*, *Ho* and *MAF* were higher for the BY population compared with the DQ population. Analysis of the genetic diversity of tea plant populations from four different species revealed that *Pi *of the *C. gymnogyna* population was the highest, while *Pi *of the *C. sinensis* population was the lowest, and *Fis *of *C. tachangensis* population was significantly higher compared with that of *C. sinensis*, *C. gymnogyna* and near *C. taliensis* populations. *Ho* of the near *C. taliensis* population was significantly higher than that of *C. gymnogyna*, *C. tachangensis* and *C. sinensis* populations (Additional file 2: Table S4).

**Fig. 2:**
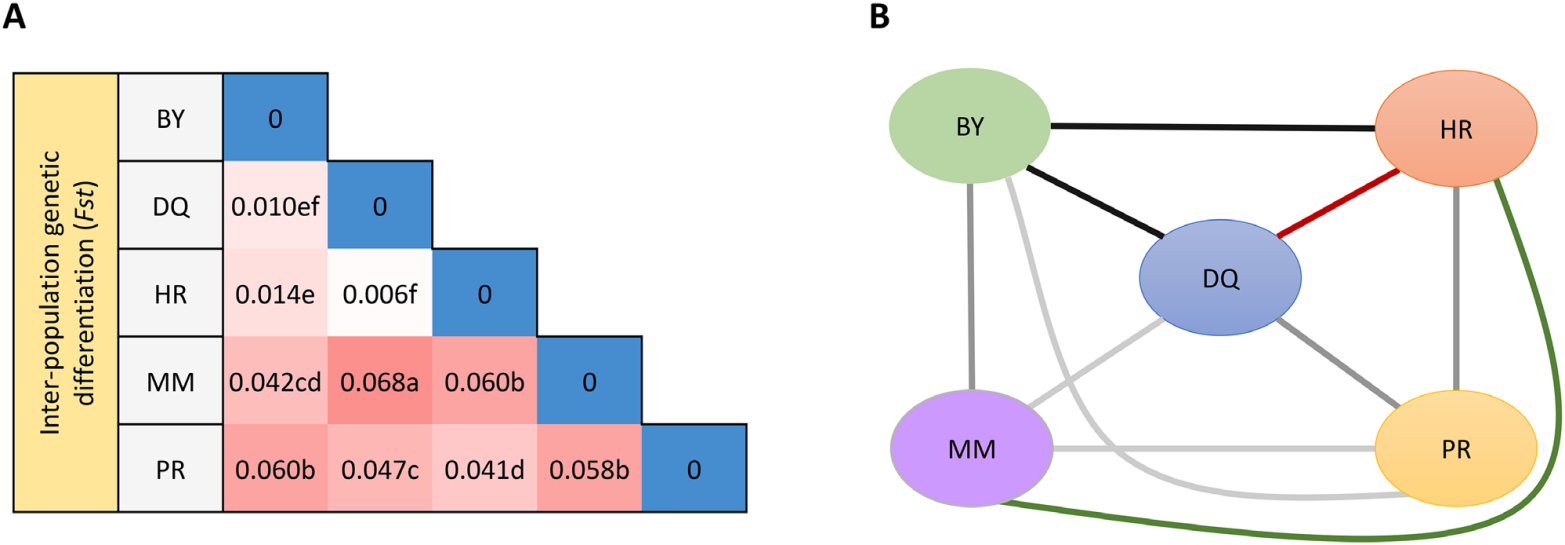
Pairwise *Fst* and genetic distance among five indigenous communities of 415 accessions in Guizhou Plateau. (**A**) Inter-population genetic differentiation coefficient (*Fst*). The colors and numbers in the cells of the matrix represent the *Fst* values and the different letters indicate a significant difference in *p* = 0.05 levels by the T-test. (**B**) Pairwise genetic distance (*GD*), the lines in different colors represent the value of genetic distance, with red representing the shortest distance for the others, green representing the longest, other colors with darker colors representing the shorter distance, and lighter colors representing the longer distance. Source data underlying (**B**) is provided as an additional file: 2 Table S5. (**A and B**) HR: Han indigenous communities, DQ: Tujia and Yi indigenous communities, BY: Buyi indigenous communities, MM: Miao indigenous communities, and PR: Gelao indigenous communities

Tajima’s *D* represents intraspecific polymorphism based on locus variation. The positive Tajima’s *D* values of the evaluated tea populations indicated that they all underwent balancing selection or a bottleneck, and populations suddenly shrank [34] (Table 1 and Additional file 2: Table S4). The genetic differentiation coefficient (*Fst*) is used as a measure of population structure, and *Fst* of 0–0.05, 0.05–0.15, 0.15–0.25 and above 0.25 indicate small, moderate, large and great divergence, respectively [33]. In this study, *Fst* of indigenous community populations ranged from 0.006 to 0.068, with an average value of 0.041. The maximum *Fst* value (0.068) was observed in DQ vs. MM, indicating that there was moderate divergence between these two populations. In addition, the minimum *Fst* value (0.006) was observed in DQ vs. HR, indicating that there was small divergence between these two populations (Fig. 2A). Notably, the lowest *Nm* value was recorded in DQ vs. MM, whereas the highest *Nm* value was showed in DQ vs. HR. At a geographical level, the lowest pairwise genetic distance (*GD*) was for DQ vs. HR (Fig. 2B and Additional file 2: Table S5).

**Table 1:** Genetic diversity of 415 tea accessions in Guizhou Plateau

Type		Number of accessions				Tajima’s *D*	*Pi*	*Ho*	*MAF*	*Fis*
		WA	AL	ML	Total					
Indigenous communities	MM	67	17	23	107	1.098	0.247a	0.074b	0.169a	0.714a
	PR	42	12	1	55	0.722	0.234b	0.092a	0.157b	0.617b
	BY	23	52	14	89	0.503	0.210c	0.071c	0.134c	0.676ab
	DQ	24	77	38	139	0.521	0.199d	0.070d	0.127d	0.657b
	HR	3	16	1	20	0.058	0.181e	0.053e	0.117e	0.706ab
Cultivation status	WA	-	-	-	159	1.240	0.246a	0.081a	0.170a	0.680a
	AL	-	-	-	174	0.529	0.181b	0.068b	0.118b	0.638b
	ML	-	-	-	82	0.325	0.167c	0.069b	0.109c	0.599b
All	All	159	174	82	415	1.326	0.226	0.073	0.144	0.691

*Note* nucleotide diversity (*Pi*), observed heterozygosity (*Ho*), minor allele frequency (*MAF*) and inbreeding coefficient (*Fis*). In the same type and line, the different letters indicate a significant difference in *p* = 0.05 levels by the T-test. Cultivation status includes wild type accessions (WA), ancient landraces (AL) and modern landraces (ML). Five indigenous communities include HR, DQ, BY, MM and PR indigenous communities

### Population structure analysis

We revealed the ancestral genetic components of the 415 tea accessions by different methods and inferred the population structure. This study assumed ancestral populations (*K*) ranging from 2 to 9. The optimal ancestral population (*K*) value was determined according to the cross-validation (CV) error, and the optimal value obtained in this study was *K* = 5 (Additional file 5: Fig. S5). Based on CV error, five ancestral genetic components were detected (Additional file 1: Table S2). Accessions with membership coefficients > 0.60 were assigned to the pure population, while those with membership coefficients < 0.60 were assigned to the admixture population [33]. The first pure population (referred to as the ancient landraces of DQ or GP01 hereafter) was composed of 131 (74.9%) ancient landraces, 26 (14.9%) modern landraces, and 18 (10.3%) wild type accessions, of which 154 (88.0%) belonged to *C. sinensis*, mainly from the DQ indigenous community. The second pure population (referred to as the wild type accessions of MM or GP02 hereafter) was composed of 51 wild-type *C. tachangensis *germplasms, mainly from the MM indigenous community. The third pure population (referred to as the ancient landraces of BY or GP03 hereafter) consisted of 18 (62.1%) ancient landraces, 3 (10.3%) modern landraces, and 8 (27.6%) wild type accessions, of which 21 (72.4%) belonging to *C. sinensis *were mainly from the BY indigenous community. The fourth pure population (referred to as the wild type accessions of PR or GP04 hereafter) consisted of 18 wild germplasms, including 12 (66.7%) *C. gymnogyna *and 6 (33.3%) *C. tachangensis*, mainly from the PR indigenous community. The fifth pure population (referred to as the modern landraces of BY and DQ or GP05 hereafter) was composed of 43 germplasms, of which 37 (86.0%) were modern landraces, mainly belonging to the *C. sinensis *from the BY and DQ integrate indigenous communities. Ninety-nine accessions (approximately 23.9% of a total of 415 samples) had admixture ancestry. In the admixture population (referred to as the waterway traffic population of multiethnic communities or GP06 hereafter) 64 (64.6%) were wild type accessions, 19 (19.2%) were ancient landraces, and 16 (16.2%) were modern landraces, including 33 (33.3%) *C. sinensis*, 31 (31.3%) *C. tachangensis*, 3 (3.0%) near *C. taliensis*, and 32 (32.3%) *C. gymnogyna* from the BY, HR, MM, PR and DQ indigenous communities. Based on the geographical location, among them, the origin of 78 (78.8%) accessions were near the rivers, while 21 (21.2%) accessions were far away from them, of which 14 (66.7%) accessions were near the ancient traffic post road, but 7 (33.3%) accessions were far away from them (Additional file 1: Table S3).

**Fig. 3:**
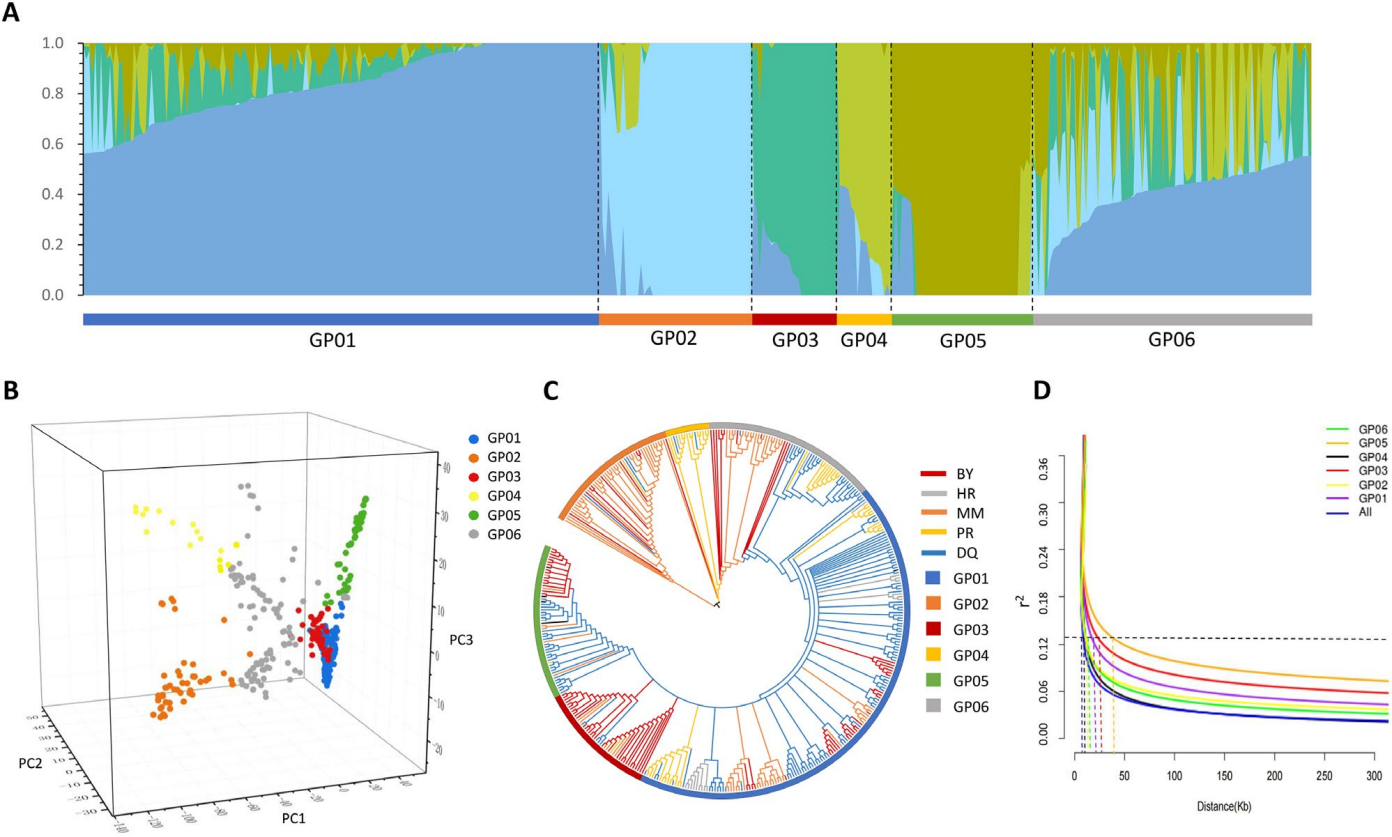
Phylogenetic relationships and population structure of 415 tea accessions. (**A**) Inferred population structure of 415 accessions. Bar plot of individual membership coefficients for the genetic clusters is inferred by using ADMIXTURE (*K* = 5) base on 99,363 SNPs. Individual membership coefficients are sorted within each cluster. (**B**) Scatter diagram of the first three principal components. The first, second and third coordinates indicate PC1, PC2 and PC3. Each dot represents an accession. (**C**) A Neighbor-Joining (NJ) phylogenetic tree of all 415 tea accessions in Guizhou Plateau constructed using whole-genome SNP data based on the maximum composite likelihood (MCL) model. The NJ tree compared with indigenous communities (HR, DQ, BY, MM and PR indigenous communities) and ADMIXTURE software results (*K* = 5). The lines with different colors represent the indigenous communities (the black blocks in the middle represent accessions imported from other provinces), and the squares with different colors nearby represent the populations we divided according to the results of ADMIXTURE. GP01, GP02, GP03, GP04 and GP05 are pure populations and GP06 is admixture population. (**D**) The LD decay plot of 415 accessions and six inferred populations

The collection of 415 accessions were analyzed by principal component analysis (PCA) and the Neighbor-Joining (NJ) phylogenetic tree to better understand the relationships between potential inferred populations. The result of PCA was highly consistent with that of the ADMIXTURE analysis (Fig. 3A, B and Additional file 5: Fig. S3). PCA revealed five main clusters, corresponding to five pure populations identified by the ADMIXTURE software. The GP02 and GP04 clusters were more scattered than GP01, GP03 and GP05, and the GP06 cluster was distributed between these five clusters along the PC1, PC2 and PC3 axes. The NJ tree also agreed with the ADMIXTURE analysis results (Fig. 3C). Furthermore, the results of the NJ tree were highly consistent with those of indigenous communities (MM, PR, BY, DQ and HR), the cultivation status (WA, AL and ML), and the species classification (*C. sinensis*, *C. tachangensis*, near *C. taliensis* and *C. gymnogyna*) (Additional file 5: Fig. S6).

### Linkage disequilibrium analysis

In this study, 29,393,327 non linkage disequilibrium (LD) pruned SNPs were used to estimate the LD of 415 tea accessions. LD decay occurred rapidly with the increase in physical distance. The maximum *r*^*2*^ value was 0.25 for the LD decay in all 415 tea accessions. As LD decayed to its half maximum (*r*^*2*^ = 0.13), the corresponding physical distance was approximately 8 Kb (Fig. 3D).

LD decay in these six inferred populations was estimated. The lowest LD decay was observed in GP05, as *r*^*2*^ reached 0.13 (threshold) at approximately 40 Kb. On the contrary, LD decay was the highest in GP04, with *r*^*2*^ = 0.13 at the corresponding physical distance of approximately 12 Kb. The corresponding physical distances of GP02, GP06, GP01 and GP03 at *r*^*2*^ = 0.13 were approximately 15 Kb, 17 Kb, 26 Kb and 31 Kb, respectively.

**Fig. 4:**
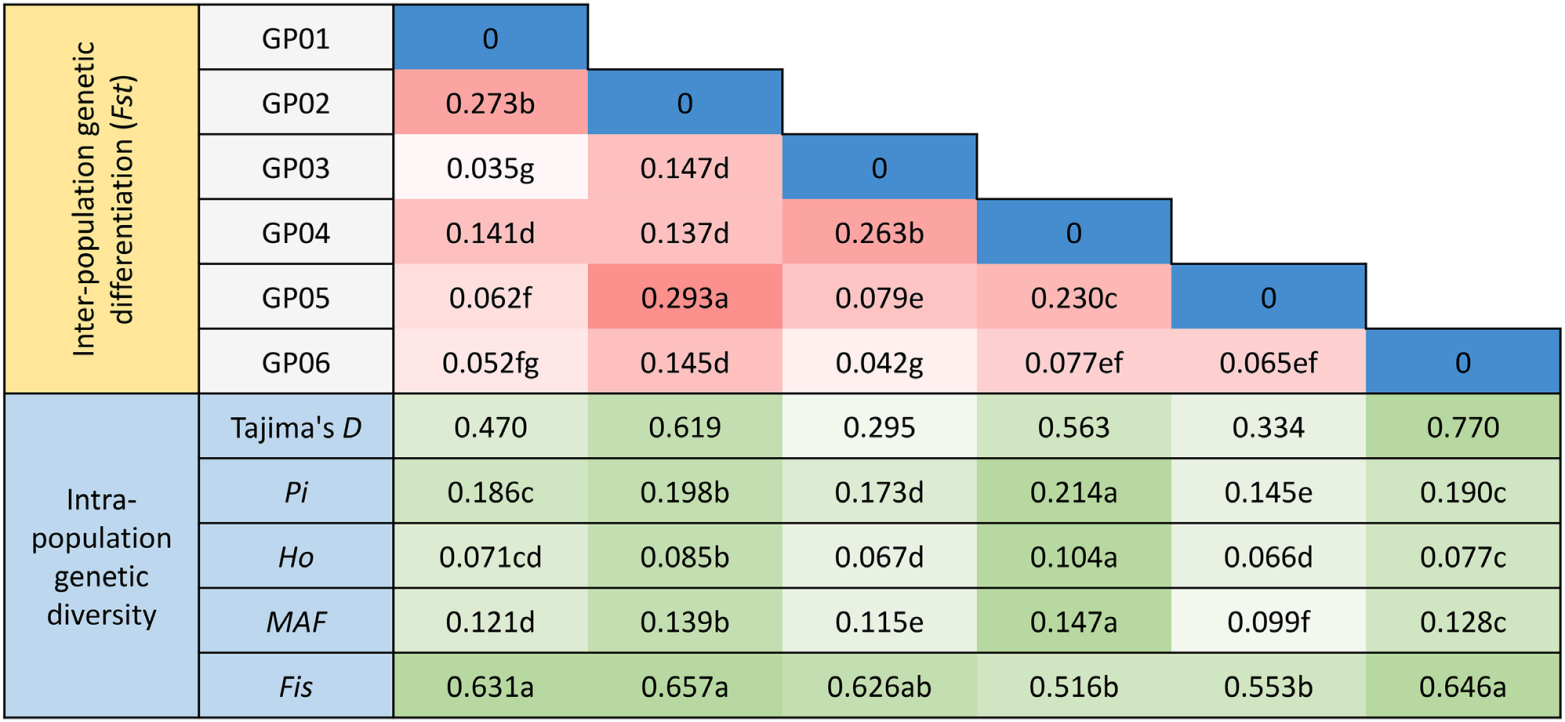
Genetic diversity of six inferred populations of 415 accessions in Guizhou Plateau. Inter-population genetic differentiation coefficient (*Fst*), nucleotide diversity (*Pi*), observed heterozygosity (*Ho*), minor allele frequency (*MAF*) and inbreeding coefficients (*Fis*). The colors and numbers in the cells of the matrix represent the *Fst* values. The colors and numbers in the cells below the *Fst* matrix represent the genetic diversity indices. The different letters indicate a significant difference in *p* = 0.05 levels by the T-test. GP01, GP02, GP03, GP04 and GP05 are pure populations and GP06 is admixture population base on ADMIXTURE software at *K* = 5

### Genetic differentiation analysis of the inferred populations

According to the results of population structure analysis, the genetic diversity indices were calculated for GP01, GP02, GP03, GP04, GP05 and GP06. *Pi*, *Ho* and *MAF* for GP04 were the highest, while those for GP05 were the lowest. We also found that the *Fis* was the highest in GP02 but the lowest in GP04. The Tajima’s *D* values in all six inferred populations were positive, indicating the occurrence of balancing selection or population bottlenecks (Fig. 4).

The *Fst* values in inferred populations ranged from 0.035 to 0.293, with an average value of 0.136. (Fig. 4). The maximum *Fst* value (0.293) was observed in GP02 vs. GP05, indicating that there was the great divergence between these two populations. The minimum *Fst* value (0.035), however, was observed in GP01 vs. GP03, indicating that there was the small divergence between these two populations. Moreover, the highest *Nm* value was showed in GP01 vs. GP03, which corresponded to the lowest pairwise *GD* value (Additional file 2: Table S6).

### Core collection development

Core collections provide GenBank curators and plant breeders with a way to reduce the size of their collections and populations while minimizing the impact on genetic diversity and allele frequency. This information can be used in molecular marker-assisted breeding, GWAS and other applications [35, 36]. We used a simple and flexible method, Core Hunter 3, to construct a core collection of 136 accessions (~ 1/3 of the original population size), which were selected to represent a set of 415 tea accessions and henceforth referred to as the ‘core set’ (Additional file1: Table S1).

**Fig. 5:**
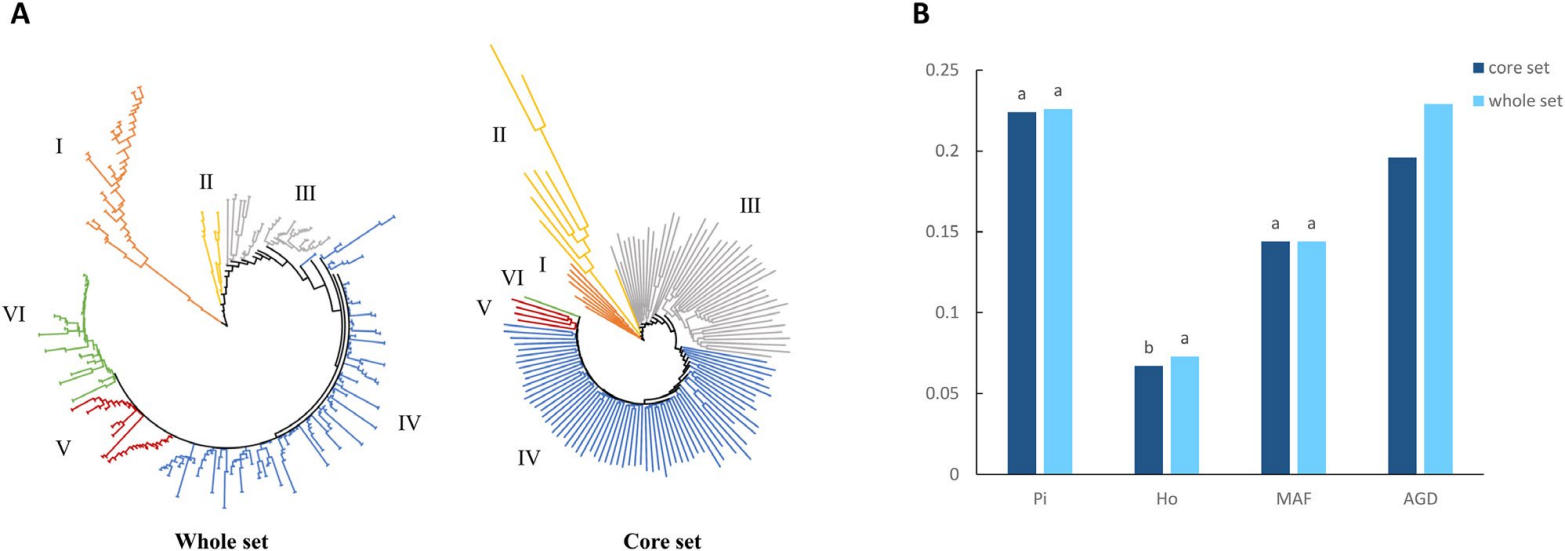
Construction a core collection of 415 tea accessions population. (**A**) The NJ trees of whole set and core set. (**B**) Genetic diversity of whole and core sets. Nucleotide diversity (*Pi*), observed heterozygosity (*Ho*), minor allele frequency (*MAF*) and average genetic distance (*AGD*). The different letters indicate a significant difference in *p* = 0.05 levels by the T-test

To evaluate the quality of the core set, we constructed the NJ trees and used the *GD* matrix to verify whether its backbone changed. We found that the core accessions were evenly distributed across the whole collection of 415 accessions that were consistently revealed by the NJ trees (Fig. 5A). We evaluated *Pi*, *Ho*, *MAF* and *GD* among the whole set and the core set. The core set had 100% of *MAF*, 99% of *Pi*, and 92% of *Ho* of the whole set. The average genetic distance (*AGD*) for the whole and core sets were 0.229 and 0.196, respectively (Fig. 5B). In the *GD* matrix of the whole set and the core set, the range of *GD* frequency (0.1 to 0.3) was the highest (Additional file 5: Fig. S7 A). These results showed that the core set included WA, AL and ML from five indigenous communities (Additional file 5: Fig. S7 B and C). Therefore, they could represent the genetic diversity of the whole set.

### Phenotypic variation analysis of OTL and OtL

The OTL and OtL traits of 252 tea accessions were investigated. Table 2 showed that the average values of OTL and OtL were 4.325 and 5.944, with a coefficient of variation (CV) of 35.2% and 23.0%, respectively. Extensive genetic variation in OTL and OtL was investigated, and the phenotypic values of the OTL and OtL traits showed normal distribution or approximately normal distribution (Additional file 5: Fig. S8 A and B). These results revealed a broad trait diversity among the populations of 252 tea accessions.

**Table 2:** Statistical analysis of OTL and OtL

Trait	Mean	Std.dev	Minimum	Maximum	Skewness	Skewness (Std.Err)	Kurtosis	Kurtosis (Std.Err)	CV (%)
OTL	4.325	1.522	1	7	-0.084	0.153	-0.319	0.306	35.2
OtL	5.944	1.370	2	9	-0.527	0.153	0.845	0.306	23.0

### GWAS analysis for OTL and OtL

**Fig. 6:**
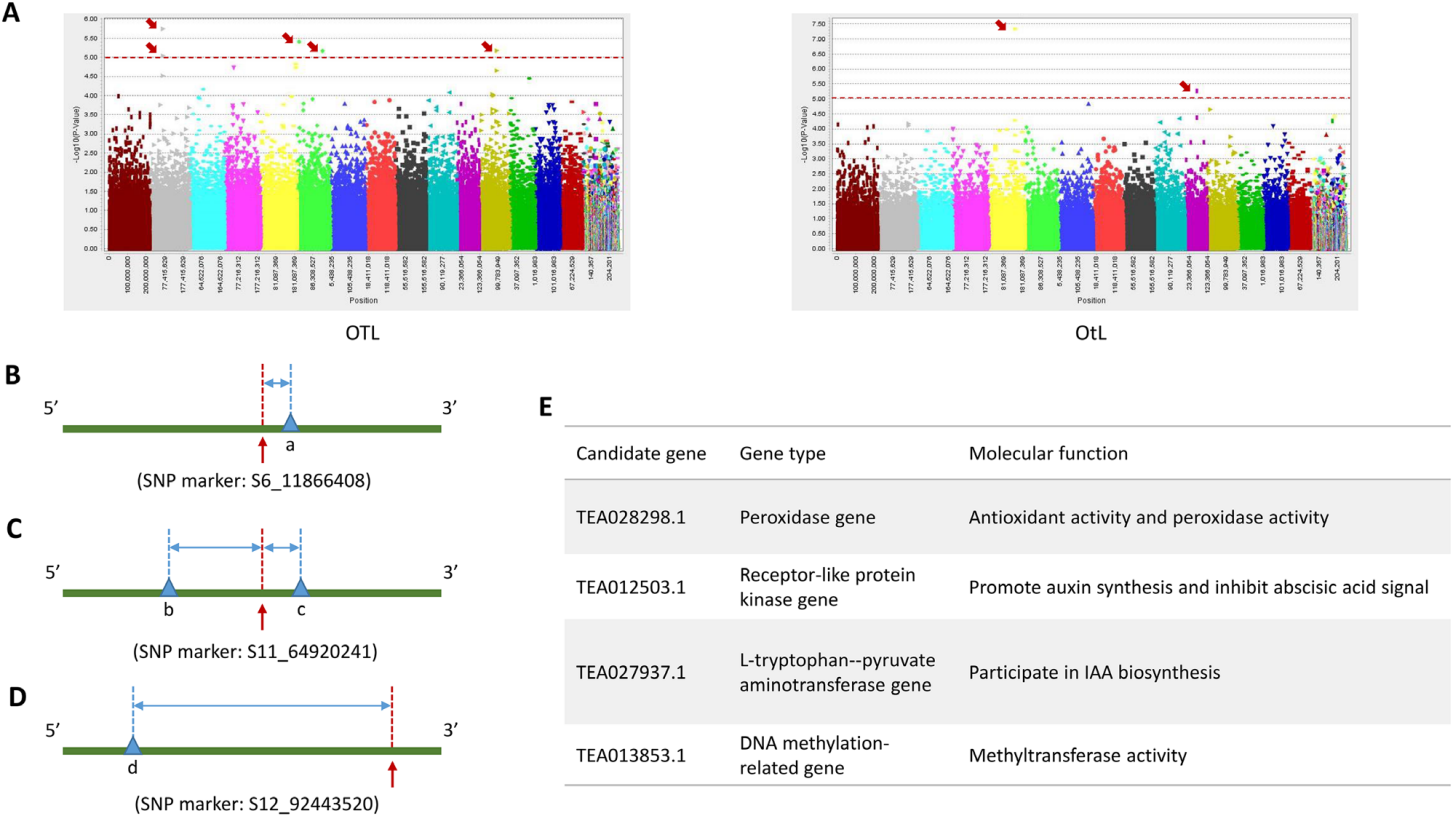
GWAS analysis and discovery of candidate genes for OTL and OtL. (**A**) Manhattan plot for the OTL of tea plant by the optimal model of GLM, and Manhattan plot for the OtL of tea plant by the optimal model of MLM. The red dashed horizontal line indicates the significance threshold (− log_10_(*P*) is about equal to 5.00). (**B, C and D**) Genes within 50 Kb regions surrounding the significant trait-associated SNP loci according to BLAST analysis using the tea plant reference genome. (**B**) a is peroxidase gene, 1.79 Kb. (**C**) b is L-tryptophan–pyruvate aminotransferase gene, 8.30 Kb. c is DNA methylation-related gene, 2.39 Kb. (**D**) d is receptor-like protein kinase gene, 27.03 Kb. (**E**) Molecular function annotations of candidate genes

The MLM and GLM methods were used for performing GWAS to detect and evaluate the genotypic variations in OTL and OtL of tea plant. In addition, the QQ plots were used to assess the extent of the difference between the observed and the expected *P*-values (Additional file 5: Fig. S9 A and B). The GLM method found a total of five SNP markers significantly associated with OTL (− log_10_(*P*) = 5.00, *P* = 1/99,363), including S2_68972914 with *R*^*2*^ = 14.19% and allele T/A, S6_11866408 with *R*^*2*^ = 12.12% and allele C/G, S12_92443520 with *R*^*2*^ = 13.75% and allele G/T, S6_137947647 with *R*^*2*^ = 11.94% and allele C/T, and S2_68972933 with *R*^*2*^ = 12.02% and allele G/T. The MLM method found two SNP markers significantly associated with OtL (− log_10_(*P*) = 5.00), including S5_141340508 and S11_64920241 with *R*^*2*^ = 22.44% and allele T/C, and *R*^*2*^ = 12.94% and allele C/T, respectively (Table 3). The Manhattan plots were shown in Fig. 6A.

Making further efforts to reveal the functions of the trait-associated SNPs, we searched for genes located within the 50 Kb regions surrounding the SNP loci using the BLAST analysis by the tea plant reference genome [31]. Finally, we found two genes located within the candidate regions probably associated with OTL, including a receptor-like protein kinase gene and a peroxidase gene. We also found two genes located within the candidate regions probably associated with OtL, including an L-tryptophan–pyruvate aminotransferase gene and a DNA methylation-related gene (Fig. 6B–E).

**Table 3:** SNP loci significantly associated with OTL and OtL using GLM and MLM

Trait	SNP Marker	Chromosome	Position	−log_10_*P*	*R*^2^(%)	Allele
OTL	S2_68972914	2	68,972,914	5.75	14.19%	T/A
	S6_11866408	6	11,866,408	5.41	12.12%	C/G
	S12_92443520	12	92,443,520	5.18	13.75%	G/T
	S6_137947647	6	137,947,647	5.17	11.94%	C/T
	S2_68972933	2	68,972,933	5.04	12.02%	G/T
OtL	S5_141340508	5	141,340,508	7.34	22.44%	T/C
	S11_64920241	11	64,920,241	5.26	12.94%	C/T

*Note* Significant SNP loci with − log_10_(*P*) = 5.00 (*P* = 1/n, *n* = 99,363). *R*^*2*^ is the percentage of phenotypic variance explained by the SNP

### RT-qPCR analysis

**Fig. 7:**
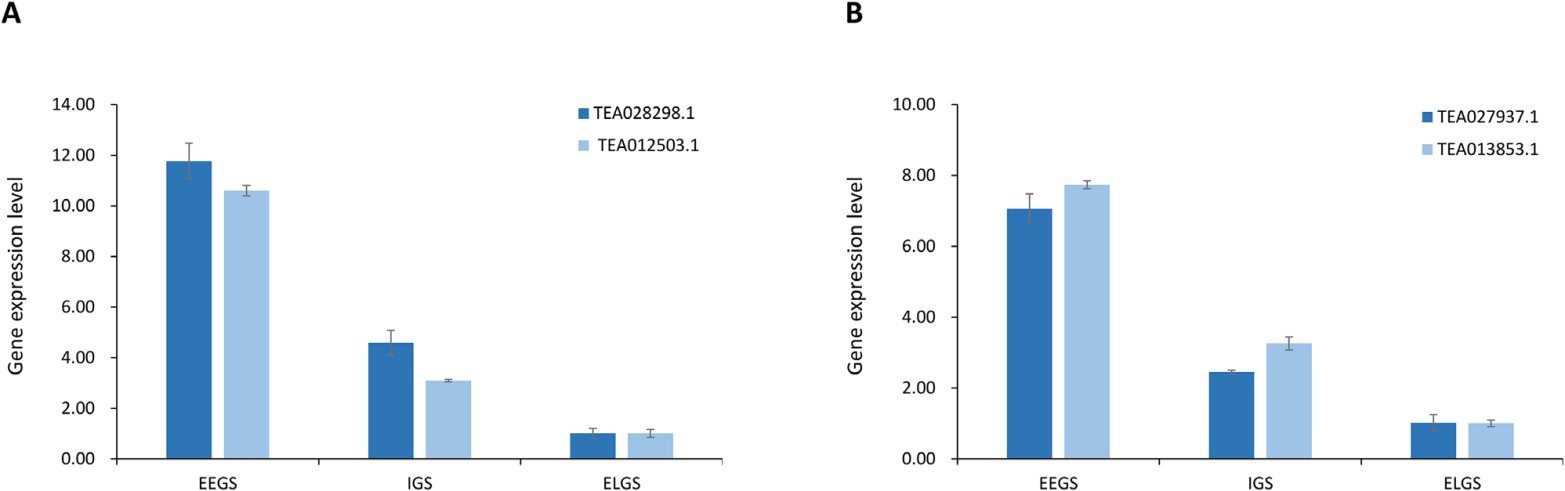
RT-qPCR analysis of four candidate genes for OTL and OtL. (**A**) The expression level of TEA028298.1 and TEA012503.1 genes, two candidate genes associated with OTL. (**B**) The expression level of TEA027937.1 and TEA013853.1 genes, two candidate genes associated with OtL. The EEGS, IGS and ELGS represent the three stages of tea accessions germination period, including the extremely early germination stage (EEGS), intermediate germination stage (IGS), and extremely late germination stage (ELGS). Source data underlying is provided as an additional file 6

To determine whether four potential candidate genes control OTL and OtL of tea plant, we measured the expression levels of the TEA028298.1 and TEA012503.1 genes associated with OTL, and the TEA027937.1 and TEA013853.1 genes associated with OtL using RT-qPCR approach. The results showed that the expression levels of these potential candidate genes were different in tea accessions at three germination stages. They all had the highest expression levels in accessions at the extremely early germination stage (EEGS) (Fig. 7A and B and Additional file 6).

## Discussion

### Genetic diversity analysis

GBS has been widely used to analyze the genetic diversity of rice [37], olive [38] and Chinese chestnut (*Castanea mollissima* Blume) [39], which is a convenient and cost-effective approach [40, 41]. We used stringent filtering criteria to generate 390.30 Gb clean reads and identify 99,363 high-quality SNPs from 415 tea accessions. The number of SNPs identified in our result was higher than those found in previous studies [27, 42, 43], indicating that the GBS is a powerful method for the genetic diversity analysis of tea plant, and the sequencing data can be used for subsequent research.

In this study, the genetic diversity of the wild type accessions population was significantly higher compared with that of the modern landraces population, because breeding practices tend to reduce genetic diversity to a greater extent than domestication [44, 45]. We also found that the genetic diversity of modern landraces population was significantly lower than that of ancient landraces population. A reasonable explanation for this result was that inbreeding or continuous directional selection during the breeding process narrows the genetic base of germplasm, reduces genetic diversity, and increases the possibility of genetic drift during the domestication process.

Interestingly, we observed that the genetic diversity was significantly higher for the tea populations in MM and PR indigenous communities than for the tea populations in BY, DQ and HR indigenous communities. The genetic diversity results of the inferred populations were basically consistent with the corresponding indigenous community populations. Among the five pure inferred populations, the genetic diversity of GP04 and GP02 was significantly higher than that of GP01, GP03 and GP05.

Previous studies have revealed that the custom, socio-economic factors and management influenced the genetic diversity patterns of tea plant [6, 13]. We found that the culture of indigenous communities might be related to conservation practices of tea plant. For example, tea plant was important in the wedding ceremony of MM and they used it as the bride price to symbolize the fidelity and love between men and women [46]. Tea plant also played an important role in BY’s daily life. In tea drinking customs of BY, the ancient tea drinking methods and experiences have been inherited and developed. After long-term migration and settlement, BY lived in harmony with other indigenous peoples and often made friends through tea [46]. In addition, tea plant was regarded as one of the most important offerings in the ceremony of deity worship by PR [47]. Moreover, tree worship, especially tea plant, was one of the most important forms of worship for DQ. Tea plants have played an important role in the life of Yi as a tribute to ancestors whom they worship, as well as for farming practices [48].

MM and PR were the oldest ethnic groups living on the Guizhou Plateau [26, 46]. They relied more on natural resources for survival and were not good at changing the natural environment. Therefore, the natural evolutionary habits of wild tea resources have been preserved, indicating that the wild tea populations (GP02 and GP04) have abundant genetic diversity, which will facilitate the genetic improvement of tea plant in future plant breeding programs. As the two ethnic groups gradually migrated, some relatively enlightened MM evolved into BY, while some relatively enlightened PR evolved into DQ [26, 49]. The BY and DQ have developed agricultural civilization to a certain extent and have made certain choices in the utilization of tea plant [46]. The tea plants distributed in these two indigenous communities were gradually evolving towards ancient landraces, reducing the genetic diversity of the tea populations (GP03 and GP01).

Since the Tang Dynasty, HR have migrated from the northern Guizhou Plateau to the central region, which has begun to influence the culture and management of PR, DQ, BY and MM indigenous communities [49]. The integration of HR with other indigenous peoples has further developed more advanced agricultural civilization, strengthening the targeted selection and utilization of tea plant. This historical event significantly reduced the genetic diversity of modern landraces population (GP05) in ethnic fusion region. According to the geographic distribution of sample sites, many tea plant accessions were distributed at the traffic nodes along the river, which promoted frequent gene exchange among tea populations in these five indigenous communities.

### Population structure analysis

ADMIXTURE has been used to analyze the population structures of maize [50], wheat [51] and tea [52, 53]. It could effectively identify global clusters, and verified accuracy of its results via PCA and phylogenetic tree. The *K* value corresponding to the minimum CV error was deemed the optimal value for determining population structure. In this study, the clustering of 415 tea accessions correlated well with the distribution of indigenous communities at *K* = 5 using the ADMIXTURE software. A total of 415 accessions were divided into six populations, including five pure populations (GP01, GP02, GP03, GP04 and GP05) and one admixture population (GP06). The NJ tree and PCA generated the same population structure output as ADMIXTURE. GP02 was *C. tachangensis* mainly from the MM indigenous community. GP04 was *C. gymnogyna* mainly from the PR indigenous community. GP01 represented ancient landraces, *C. sinensis*, mainly from the DQ indigenous community. GP03 was ancient landraces, *C. sinensis*, mainly from the BY indigenous community. GP05 was modern landraces, *C. sinensis*, mainly from the BY and DQ integrate indigenous communities, and GP06 was an admixture population mainly distributed at traffic nodes along the river.

Previous studies have reported that the *Fst*, pairwise *GD*, *Nm* and *Fis *were the key parameters in determining genetic differentiation between populations [33, 34]. In this study, we estimated the pairwise *GD*, *Fst*, *Fis* and *Nm* among six inferred populations. The higher pairwise *GD* and *Fst *and lower *Nm* were found in GP02 vs. GP01, GP04 vs. GP03, GP02 vs. GP05, GP04 vs. GP05, GP04 vs. GP01, and GP03 vs. GP02, while the smaller pairwise *GD* and *Fst* and higher *Nm* were observed in GP03 vs.GP01, GP05 vs.GP01, and GP05 vs. GP03.

A reasonable explanation was that ethnic cultural differences have to some extent influenced the genetic differentiation of tea populations over the long term. GP02 and GP04 were wild type accessions populations, distributed in the MM indigenous community and PR indigenous community, respectively. The MM and PR were the two oldest indigenous groups living in the Guizhou Plateau [26, 46]. They lived in the high mountain jungle and had less communication with other indigenous groups, which reduced gene exchange between the tea accessions distributed in these two indigenous groups and the outside world. These two indigenous groups have always utilized natural resources rather than altering them. Therefore, these two indigenous communities maintained the higher genetic diversity and retained the primary wild type accessions. With the migration of ethnic groups, a tribe of MM gradually evolved into BY, while a tribe of PR gradually evolved into DQ [26, 49]. BY and DQ had a certain agricultural civilization. They made tea accessions further evolved into a favorable direction for utilization by selecting, thus increasing the genetic differentiation between BY (GP03) vs. MM (GP02) and DQ (GP01) vs. PR (GP04). Afterwards, with the migration of HR and the integration with BY and DQ, the development of agricultural civilization in BY and DQ was further promoted, and the selection and utilization of tea accessions were strengthened. This result further increased the genetic differentiation between the modern landraces population distributed in the BY and DQ integrate indigenous communities (GP05) and the wild type accessions populations distributed in the MM and PR indigenous communities (GP02 and GP04).

Furthermore, we found that GP06 may be a product of the gene exchange between GP04, GP02, GP01 and GP03 through transportation hubs [54, 55] and may have formed earlier than GP05. The former may be gradually distributed and evolved into GP05 due to economic trade, development of the modern tea industry and ethnical cultural exchanges. Therefore, the GP06 may have a complex evolutionary history, and its utilization as a natural resource still needs further exploration.

### Core collection development

Establishing a core collection that has the lowest level of genetic redundancy and preserves the maximum genetic diversity of the entire collection facilitates the identification of varieties suitable for GWAS [56]. Core collections can be evaluated based on different types of genetic markers, phenotypic traits and pairwise distance matrices [57]. Choosing the most appropriate evaluation method depends on the purpose of the core collection [58]. Preserving most alleles is an ideal way to conserve germplasm, while distance-based methods mainly maintain most allele combinations in specific genotypes, making them suitable for GWAS. Thus, we applied a precomputed distance matrix to GBS data to establish the core collection of tea accessions in the Guizhou plateau, including wild type accessions, ancient landraces and modern landraces from indigenous communities. The core collection was constructed based on the optimization of the average genetic distance between each accession and the nearest neighboring entry in the core collection (E-NE) model, which contained 33% of the total number of accessions. *Ho*, *Pi* and *MAF* of the core set accounted for more than 90% of the whole set, indicating that the core set can well represent 415 tea accessions for further research. This core collection will facilitate further development of GWAS and the breeding of tea varieties with excellent characteristics.

### Genome-wide association study analysis

The choice of plant materials used for association mapping is very critical as they should have a wide range of diversity to capture the maximum number of historical recombination events. Tea plant is a self-incompatible and highly heterozygous woody perennial tree. Compared with self-pollinating plants, tea plants have more extensive genetic variation and higher diversity due to domestication through hybridization, long-term allogamy, and climate-based selection [59]. We used tea populations to perform GWAS analysis and discovered a set of SNP markers and genes associated with OTL and OtL, suggesting that the offspring with high genetic diversity produced through cross-pollination are suitable for association mapping. Moreover, we found that the majority of tea accessions from DQ indigenous community were at the earlier germination stage, which may be related to the local geographical location with mild climate and sufficient rainfall [60].

In this study, we conducted a GBS-based GWAS to discover five and two SNP loci associated with OTL and OtL through the optimal GLM and MLM models, respectively, which are the two most commonly used algorithms in GWAS [61]. These associated SNP loci could be considered as the candidate genetic markers affecting OTL and OtL traits. Using BLAST with the tea plant reference genome [31], we identified two potential candidate genes related to OTL, including a receptor-like protein kinase gene and a peroxidase gene, and two potential candidate genes related to OtL, including an L-tryptophan–pyruvate aminotransferase gene and a DNA methylation-related gene within a 50 Kb region around the significant trait-associated SNP loci, which were validated using RT-qPCR approach. We found that LD became too low at distances greater than 50 Kb. Therefore, 50 Kb was selected as a reasonable distance that caused moderate LD between the gene and trait-associated SNPs [59].

Peroxidases are a superfamily of antioxidant enzymes that catalyze the oxidation of various substrates using H_2_O_2_ or other organic hydroperoxides enzymes and protect plant cells from oxidative stress damage. Evidence has proved that peroxidases not only are important components of plant growth and development but also can loosen plant cell walls, leading to dormancy break and bud germination [62]. We discovered one peroxidase gene located within 1.79 Kb downside of the significant trait-associated SNP, speculating that this gene was a potential candidate gene for the OTL trait in tea plant. The receptor-like protein kinase FERONIA is a key regulator of stress responses mediated by brassinosteroids (BRs). Previous study has shown an antagonistic relationship between ethylene and endogenous BRs for controlling hypocotyl elongation. The *fer-2* mutant exhibited the loss of responses modulated by BRs, which led to an imbalance in this relationship and increased the effect of ethylene on growth inhibition [63]. Besides, the pathway related to the FER receptor kinase has been identified, which negatively regulated the ABA response through the activation of an A-type PP2C phosphatase, ABI2, promoting plant growth and leaf development [64]. In our research, one receptor-like protein kinase gene was located within 27.03 Kb upside of the significant trait-associated SNP.

L-tryptophan aminotransferase has been proved to be involved in the biosynthesis of auxin (IAA), and its first step is catalyzed in the IPA branch of the IAA biosynthesis pathway to initiate multiple growth changes in response to environmental and developmental cues. Maintaining appropriate IAA levels in roots requires TAA1 and TAR2, while the development of proper embryo patterning requires functions of TAA1, TAR1 and TAR2, which have an impact on plant growth and leaf development [65]. In our research, one L-tryptophan–pyruvate aminotransferase gene was located within 8.30 Kb upside of the significant trait-associated SNP. RNA-directed DNA methylation (RdDM) is a process in which RNA molecules direct the addition of DNA methylation through RNA-DNA sequence interactions. Previous study has reported that the phenomenon of mRNA degradation directed by RdDM and siRNA in plants is the silencing of specific genes of matching sequence by RNA interference, which has a significant impact on the regulation of plant gene expression and development [66]. In our research, one DNA methylation-related gene was located within 2.39 Kb on the downside of the significant trait-associated SNP. These reports have confirmed our findings that the four potential candidate genes identified in this study are related to OTL and OtL of tea plant, which will be beneficial for future development of molecular markers and MAS breeding.

## Conclusions

Genome-wide SNPs in tea accessions from the origin center, Guizhou Plateau, were identified in this study using GBS. These SNPs were used to analyze the population structure, genetic diversity and LD pattern of 415 accessions, which were divided into six populations, including five pure populations (ancient landraces of DQ, ancient landraces of BY, wild type accessions of MM, wild type accessions of PR and modern landraces of BY and DQ) and one admixture population (waterway traffic population of multiethnic communities). All 415 accessions exhibited high genetic diversity. The management and cultural practices of different indigenous communities in the Guizhou Plateau may play important roles in the protection and enhancement of the genetic diversity of tea plant. We developed a core set of 415 tea accessions and identified four potential candidate genes associated with OTL and OtL traits. The results of this study will help support genetic diversity conservation, the introduction of cultivars, and further research on tea plants that are valued by different indigenous peoples.

## Methods

### Plant materials

A collection of 415 tea accessions were used in this study. Based on the geographical distribution of indigenous peoples [26], the Guizhou Plateau included five indigenous communities. Miao living area is referred to as Miao Man (MM) indigenous community (included 107 accessions), Gelao living area is referred to as Pu Ren (PR) indigenous community (included 55 accessions), Buyi living area is referred to as Bai Yue (BY) indigenous community (included 89 accessions), Tujia and Yi living area is referred to as Di Qiang (DQ) indigenous community (included 139 accessions), and Han living area is referred to Han Ren (HR) indigenous community (included 20 accessions). The other five tea accessions were collected from Fujian, Zhejiang, Hunan and Guizhou Provinces and planted in the tea garden of Guiyang, China. Among the 415 tea accessions, 159 samples were identified as arbor and 256 samples were identified as shrub, according to the field investigation of tree types. Of these, 56 samples were large leaf, 204 samples were medium leaf, and 155 were small leaf. Based on the research of Niu et al. [27], wild tea plants older than 100 years and their natural offsprings are referred to as “wild type accessions (WA)”, cultivated tea plants older than 100 years are referred to as “ancient landraces (AL)”, while cultivated tea plants from tea gardens are referred to as “modern landraces (ML)”. Of these, 159 accessions were WA, 174 accessions were AL, and 82 accessions were ML. According to the classification systems of Min [67] and Chen et al. [68], 251 *C. sinensis*, 100 *C. tachangensis*, 5 near *C. taliensis* and 59 *C. gymnogyna* were identified (Additional file 1: Table S1).

Geographic information was acquired using GPS, and based on field investigations during the sampling process and Yang et al.‘s research [26], the distributions of tea accessions in the indigenous communities of Guizhou Plateau (MM, PR, DQ, BY and HR) were determined. The GIS technology was used for mapping. Professor Suzhen Niu from the Key Laboratory of Plant Resources Conservation and Germplasm Innovation in Mountainous Region (Ministry of Education), Institute of Agro-Bioengineering, Guizhou University, associate professor Jie Yin and lecturer Qinfei Song from the College of Tea Science of Guizhou University, and researcher Zhengwu Chen from lnstitute of Tea, Guizhou Academy of Agricultural Sciences conducted morphological identification on all tea accessions, and stored them in the tea germplasm resource garden of Guizhou University. This study and experiment complied with local legislation, and national and international guidelines. The authors also complied with the Convention on the Trade in Endangered Species of Wild Fauna and Flora and Regulations of Guizhou Province on the protection of ancient tea plants.

### DNA extraction, library preparation and sequencing

DNA was extracted from young leaves using a Plant Genomic DNA Rapid Extraction Kit (Beijing Biomed Gene Technology Co., Ltd., Beijing, China). The integrity and purity of DNA were tested by electrophoresis in 1% agarose gel and the Qubit Fluorometer (Invitrogen), and then the DNA samples were stored at − 20 °C. The DNA content for library preparation was 100 ng, and the purified DNA was digested by the SacI and MseI restriction enzymes from New England Biolabs (NEB). Thereafter, the SacAD and MseAD adaptors were ligated to the end of digested DNA fragments for barcode ligation, gel DNA fragment selection, adaptor connection, and PCR amplification of fragments, and 500–550 bp amplified products were recovered. The paired-end 150 bp (PE150) sequencing was performed on the Illumina HiSeq X Ten platform [33, 34].

### SNP calling and quality control

The adaptor was trimmed with the special Perl script, and the barcodes were used to demultiplex a set of raw sequence DNA reads. The reads with a base quality value below 5 were filtered out, and the remaining reads were used as clean data, which were then mapped to the reference genome (http://tpia.teaplants.cn/) [31] using BWA-MEM (v. 0.7.10) run with the default parameters [69]. GATK (v. 3.7.0) was used to perform SNP calling [70]. The criteria for filtering SNPs were as follows: (1) The variants (SNPs) must be biallelic. (2) GATK (v. 3.7.0) was used to filter SNPs with the parameters, including QUAL < 50.0 || QD < 2.0 || FS > 60.0 || MQ < 40.0 || Mapping Quality Rank Sum (MQRankSum) < − 12.5 || ReadPosRankSum < − 8.0. (3) The VCFtools program package (v. 0.1.15) was used to filter SNPs with minor allele frequencies (MAF) < 0.05 or missing data rates > 20% [27, 71]. The CMplot (v. 3.7.0) software was used to draw the SNP density plot [33]. A total of 99,363 high-quality SNPs (through LD pruned) from the 415 tea accessions were selected and subjected to the subsequent population structure and genetic diversity analysis.

### Genetic diversity analysis

Genetic diversity indices included observed heterozygosity (*Ho*), minor allele frequency (*MAF*), inbreeding coefficient (*Fis*), nucleotide diversity (*Pi*), Tajima’s *D*, genetic differentiation coefficient (*Fst*), gene flow (*Nm*) and genetic distance (*GD*). *MAF*, *Ho* and *Fis* values of each inferred population were calculated using Plink (v. 1.9) [72]. *Pi* and Tajima’s *D* values of each inferred population and *Fst* values of the pairwise inferred populations were calculated using VCFtools (v. 0.1.15) [71]. *Nm* was calculated using the formula *Nm* = (1-*Fst*)/4*Fst* [33]. *GD* values of pairwise inferred populations were calculated by the MEGA (v. 10.2.4) software [73]. The significant differences between these indices were determined by SPSS Statistics (v. 26) software.

### Population structure analysis

PLINK (v. 1.9) was used to convert the variant call format (VCF) files into the pedigree files [72]. Population structure was analyzed by the ADMIXTURE (v. 1.3.0) software, with the number of ancestral populations (*K*) ranging from 2 to 9 [74]. To determine the optimal population structure classification, the optimal *K* value was identified based on the minimum cross-validation (CV) error [34]. The threshold value for the membership coefficient was set to 0.6 to distinguish between admixture and pure populations [33]. The TASSEL (v. 5.2.72) software was used to perform PCA [75]. To clarify the genetic relationship among the inferred populations, phylogenetic trees were constructed. MEGA (v. 10.2.4) was used to calculate the distance matrix and construct the Neighbor-Joining (NJ) phylogenetic tree based on the maximum composite likelihood (MCL) model with 1000 bootstrap replicates [76].

### Linkage disequilibrium analysis and core collection development

To estimate and compare the patterns of linkage disequilibrium (LD) between different inferred populations, the squared correlation coefficient (*r*^*2*^) between genome-wide unpruned SNPs was calculated and plotted using the PopLDdecay (v. 3.29) package [77].

We defined a core set of 415 tea accessions using the Core Hunter 3 program [57], according to the optimization of average genetic distance as described by Odong et al. [58] (Additional file 3).

### Phenotypic trait evaluation and statistical analysis

We evaluated two traits related to the germination period, including the germination period of one bud and two leaves (OTL) and the germination period of one bud and three leaves (OtL). As shown in Additional file 4, each accession was examined for signs of germination from the beginning of March until beginning of May and scored from stage 1 to 9. The TASSEL (v. 5.2.72) software [75] was used to analyze phenotypic data. Analysis of statistics was estimated by SPSS Statistics (v. 26) software based on the trait means of each tea accession.

### GWAS analysis for OTL and OtL

Based on the selected SNP markers developed from a total of 252 accessions, GWAS was performed for the OTL and OtL traits using the TASSEL (v. 5.2.72) software by two methods, namely the mixed linear model (MLM) and the general linear model (GLM) [59]. The red dotted line indicated the genome-wide control threshold − log_10_(*P*-value) = 5.00 (*P*-value was equivalent to 1/n, where n was the total number of markers applied) [59]. Manhattan and Quantile − Quantile plots (QQ plots) were drawn using TASSEL (v. 5.2.72) software [75]. The trait-associated SNP markers were obtained using the optimal GLM and MLM models. Genes located within the 50 kb regions surrounding the trait-associated SNP loci were considered potential candidate genes.

### RNA extraction for RT-qPCR analysis

We selected nine tea plant accessions, including three accessions at the extremely early germination stage (EEGS), three accessions at the intermediate germination stage (IGS), and three accessions at the extremely late germination stage (ELGS). Total RNA was extracted from each tea accession based on the traits (OTL and OtL) using the UNIQ-10 column Trizol Total RNA Purification Kit (Sangon Biotech Co., Ltd., Shanghai, China). The expression patterns of four potential candidate genes identified through GWAS analysis were verified using RT-qPCR, which was performed using the ChamQ Universal SYBR qPCR Master Mix Kit (Nanjing Novozan Biotechnology Co., Ltd., Nanjing, China). The results were analyzed using the 2^−(△△Ct)^ method [78] with GAPDH used as an internal reference gene. A total of three biological and three technical replicates were used.

### Electronic supplementary material

Below is the link to the electronic supplementary material.

Additional file 1: table S1. Information of 415 tea accessions used in this study. Table S2. Statistics of the number and ratio of the accessions of cultivation status, species classification and indigenous communities in six inferred populations. Table S3. Statistics of the number and ratio of the accessions of near ancient hubs and near rivers in six inferred populations.

Additional file 2: table S1. The quality control data of 415 tea accessions. Table S2. Statistics of heterozygosity rate of 99,363 SNPs in 415 tea accessions.Table S3. Percentage of transition and transversion SNPs identified using genotyping-by-sequencing. Table S4. Genetic diversity of 415 tea accessions in Guizhou Plateau. Table S5. The Nm and pairwise genetic distance among five indigenous community populations. Table S6. The Nm and pairwise genetic distance among six inferred populations of 415 accessions.

Additional file 3: table S1. Precomputed pairwise genetic distance matrix of 415 accessions.

Additional file 4: table S1. Phenotypic data for OTL and OtL of tea plant.

Additional file 5: fig. S1. Geographic distribution of sampling sites. Geographical distribution of Ming Dynasty ancient transportation hubs and rivers in Guizhou Plateau. Fig. S2 Geographical distribution of tea accessions collected in this study. Geographical distribution of cultivation status (i.e., wild type accessions (WA), ancient landraces (AL) and modern landraces (ML)) and species classification (i.e., *C. sinensis*, *C. tachangensis*, near *C. taliensis* and *C. gymnogyna*) in Guizhou Plateau. Fig. S3 Geographic distribution of sampling sites. Geographical distribution of populations (i.e., GP01, GP02, GP03, GP04, GP05 and GP06) inferred by ADMIXTURE software (*K* = 5) in Guizhou Plateau. Fig.  S4. Distribution map of SNPs on 15 chromosomes graph. Fig. S5. Graph for CV error in the range of *K* = 2–9 of 415 tea accessions. Fig. S6. Cluster analysis using NJ trees. Fig. S7. Summary of comparison information among core and whole sets. Fig. S8. Phenotype frequency distribution of OTL and OtL. Fig. S9.GWAS analysis for OTL and OtL.

Additional file 6: table S1. Analysis of TEA028298.1 potential candidate gene expression in 9 tea accessions. Table S2. Analysis of TEA012503.1 potential candidate gene expression in 9 tea accessions. Table S3. Analysis of TEA027937.1 potential candidate gene expression in 9 tea accessions. Table S4. Analysis of TEA013853.1 potential candidate gene expression in 9 tea accessions.

## Data Availability

The plant materials are growing in our resource nursery and are available from the corresponding author on reasonable request. The raw sequence data reported in this study have been deposited in the Genome Sequence Archive [79] in BIG Data Center, Beijing Institute of Genomics (BIG), Chinese Academy of Sciences, under accession number CRA001438 that is publicly accessible at http://bigd.big.ac.cn/gsa.

## Abbreviations

*C. sinensis*: *Camellia sinensis* (L.) O. Kuntze
*C. gymnogyna*: *Camellia gymnogyna* Chang
*C. tachangensis*: *Camellia tachangensis* F.C. Zhang
*C. taliensis*: *Camellia taliensis* (W.W. Smith) Melchior
GBS: Genotyping-by-sequencing
GWAS: Genome-wide association study
SNPs: Single-nucleotide polymorphisms
OTL: Germination period of one bud and two leaves
OtL: Germination period of one bud and three leaves
MM: Miao Man
PR: Pu Ren
DQ: Di Qiang
BY: Bai Yue
HR: Han Ren
LD: Linkage disequilibrium
WA: Wild type accessions
AL: Ancient landraces
ML: Modern landraces
Ho: Observed heterozygosity
MAF: Minor allele frequency
Fis: Inbreeding coefficient
Pi: nucleotide diversity
Fst: Genetic differentiation coefficient
Nm: Gene flow
GD: Genetic distance
PCA: Principal component analysis
CV error: Cross-validation error
NJ tree: Neighbor-Joining tree
MCL: Maximum composite likelihood
MLM: Mixed linear model
GLM: General linear model
EEGS: Extremely early germination stage
IGS: Intermediate germination stage
ELGS: Extremely late germination stage
AGD: Average genetic distance
E-NE: The average genetic distance between each accession and the nearest neighboring entry in the core collection
RdDM: RNA-directed DNA methylation
