# Supplementary material for: Genome-wide association study, population structure, and genetic diversity of the tea plant in Guizhou Plateau
Source: BMC Plant Biol. 2024 Jan 30;24:79. doi: 10.1186/s12870-024-04761-x (PMC10826100; doi:10.1186/s12870-024-04761-x)
Supplement: Supplementary file 5 — Additional file 5: fig. S1. Geographic distribution of sampling sites. Geographical distribution of Ming Dynasty ancient transportation hubs and rivers in Guizhou Plateau. Fig. S2 Geographical distribution of tea accessions collected in this study. Geographical distribution of cultivation status (i.e., wild type accessions (WA), ancient landraces (AL) and modern landraces (ML)) and species classification (i.e., C. sinensis, C. tachangensis, near C. taliensis and C. gymnogyna) in Guizhou Plateau. Fig. S3 Geographic distribution of sampling sites. Geographical distribution of populations (i.e., GP01, GP02, GP03, GP04, GP05 and GP06) inferred by ADMIXTURE software (K = 5) in Guizhou Plateau. Fig. S4. Distribution map of SNPs on 15 chromosomes graph. Fig. S5. Graph for CV error in the range of K = 2–9 of 415 tea accessions. Fig. S6. Cluster analysis using NJ trees. Fig. S7. Summary of comparison information among core and whole sets. Fig. S8. Phenotype frequency distribution of OTL and OtL. Fig. S9.GWAS analysis for OTL and OtL. [file 12870_2024_4761_MOESM5_ESM.docx]

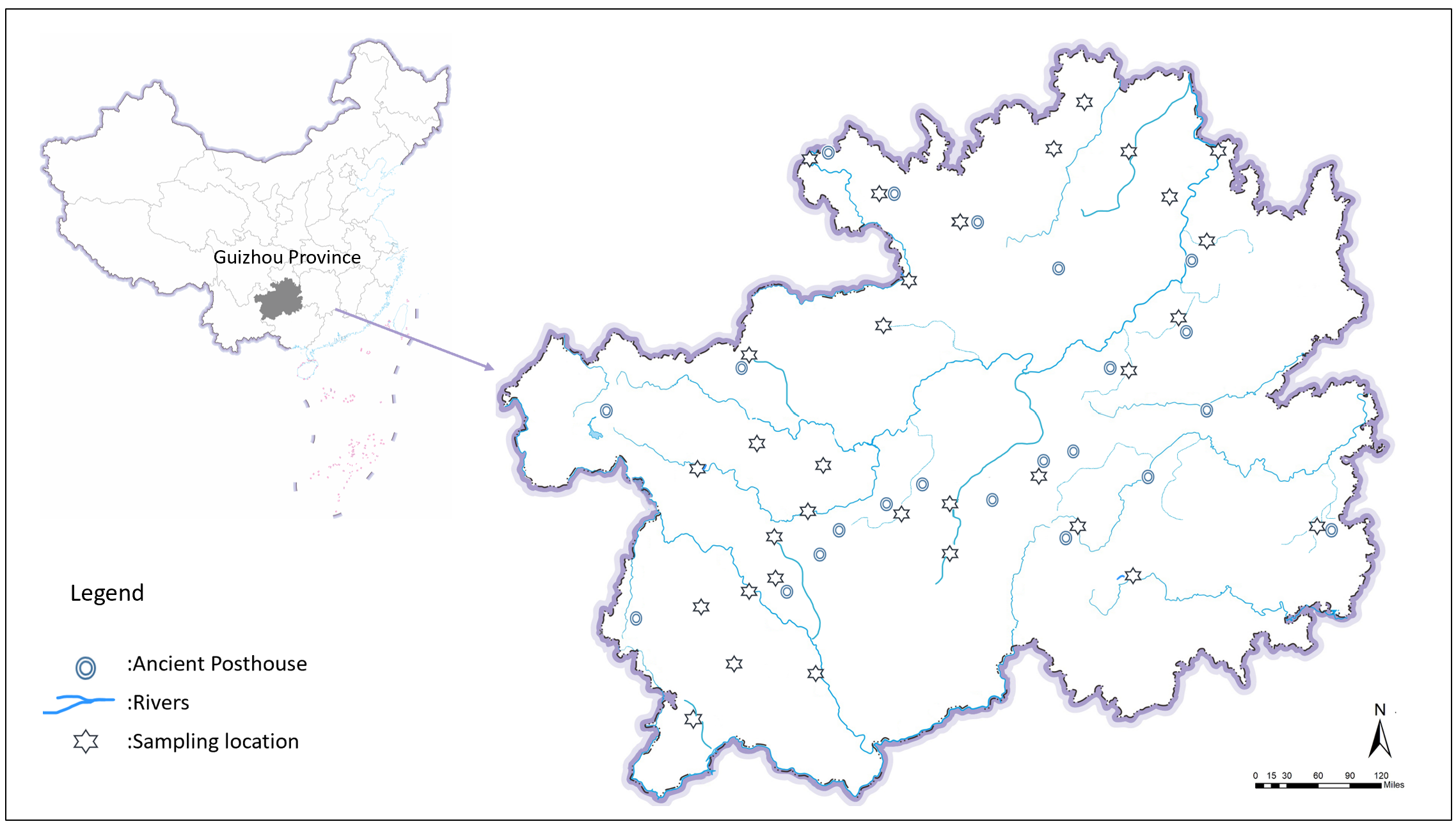


**Fig. S1** Geographic distribution of sampling sites. Geographical distribution of Ming Dynasty ancient transportation hubs and rivers in Guizhou Plateau [54, 55]. Rings represent ancient post houses. The blue bands represent rivers. Hexagonal stars represent sampling locations. The geographic data information used in the map came from field investigations, standard map services (http://bzdt.ch.mnr.gov.cn/download.html?searchText), and geographic information surveyed by the Natural Resources Department of Guizhou Province (https://zrzy.guizhou.gov.cn/wzgb/zwgk/zdlyxxgk/dlxxgl/), and the map was drawn using ArcGIS software


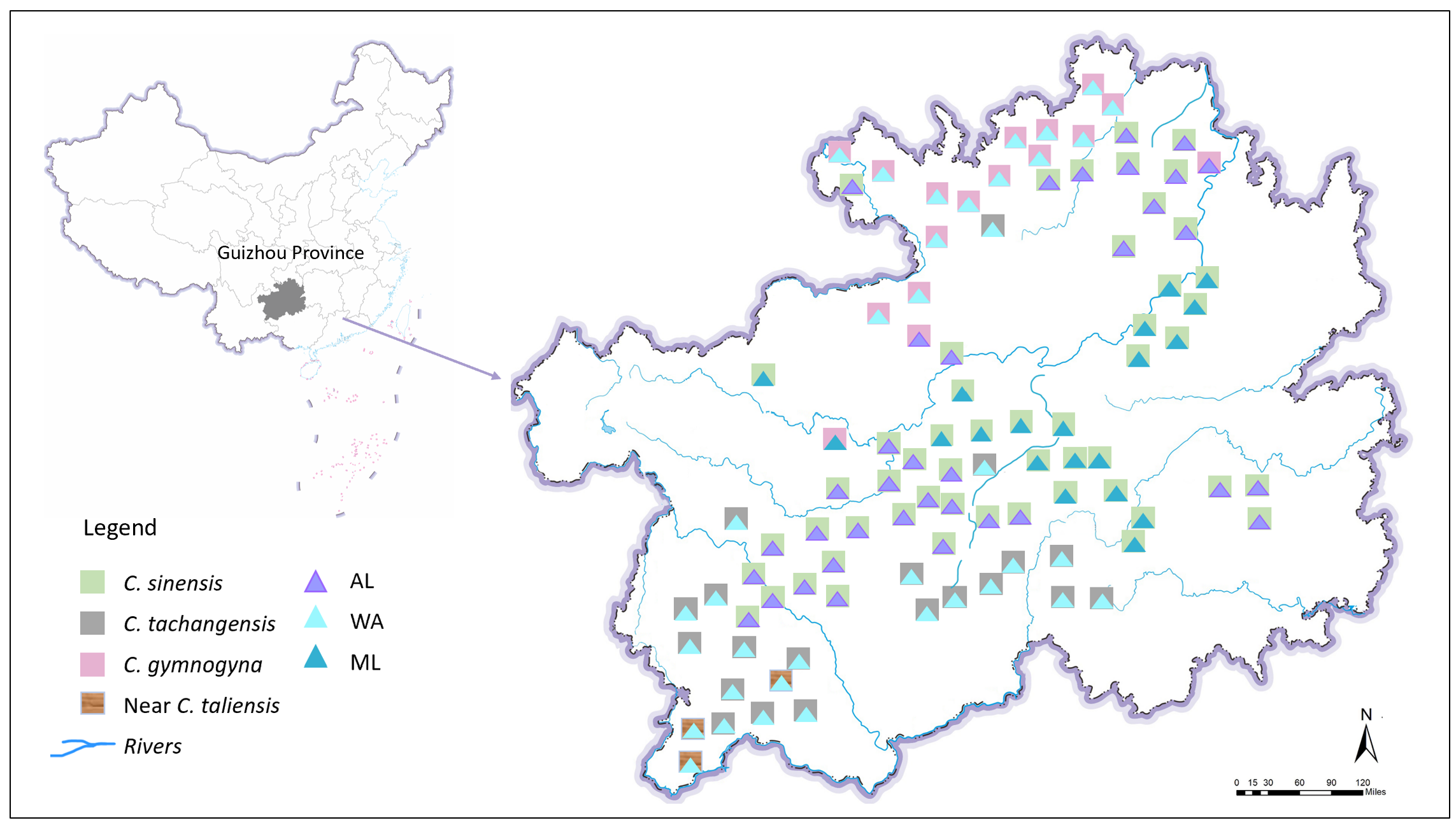


**Fig. S2** Geographical distribution of tea accessions collected in this study. Geographical distribution of cultivation status (i.e., wild type accessions (WA), ancient landraces (AL) and modern landraces (ML)) and species classification (i.e., *C. sinensis*, *C. tachangensis*, near *C. taliensis* and C. *gymnogyna*) in Guizhou Plateau. The geographic data information used in the map came from field investigations, standard map services (http://bzdt.ch.mnr.gov.cn/download.html?searchText), and geographic information surveyed by the Natural Resources Department of Guizhou Province (https://zrzy.guizhou.gov.cn/wzgb/zwgk/zdlyxxgk/dlxxgl/), and the map was drawn using ArcGIS software


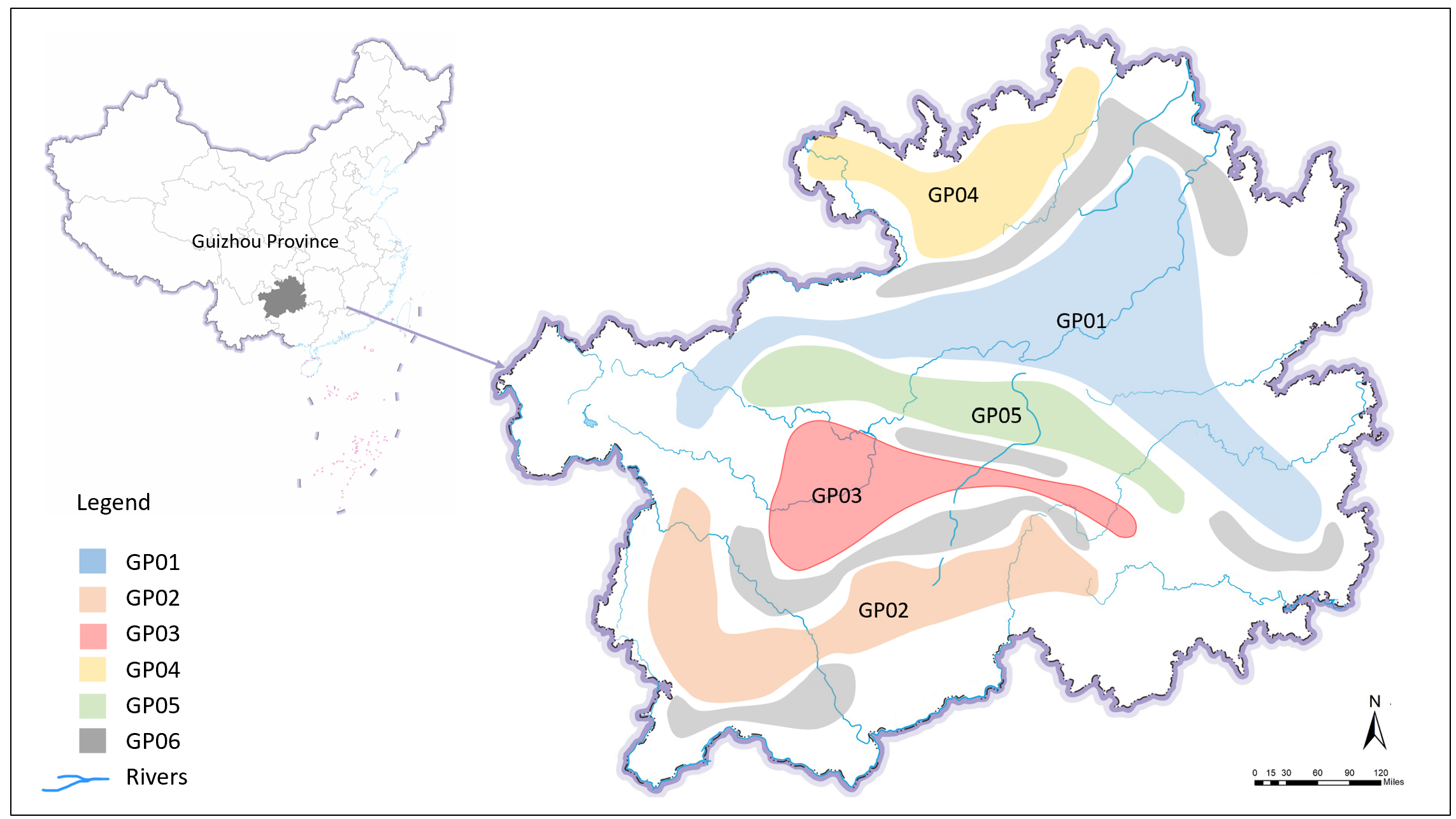


**Fig. S3** Geographic distribution of sampling sites. Geographical distribution of populations (i.e., GP01, GP02, GP03, GP04, GP05 and GP06) inferred by ADMIXTURE software (*K*=5) in Guizhou Plateau. The geographic data information used in the map came from field investigations, standard map services (http://bzdt.ch.mnr.gov.cn/download.html?searchText), and geographic information surveyed by the Natural Resources Department of Guizhou Province (https://zrzy.guizhou.gov.cn/wzgb/zwgk/zdlyxxgk/dlxxgl/), and the map was drawn using ArcGIS software


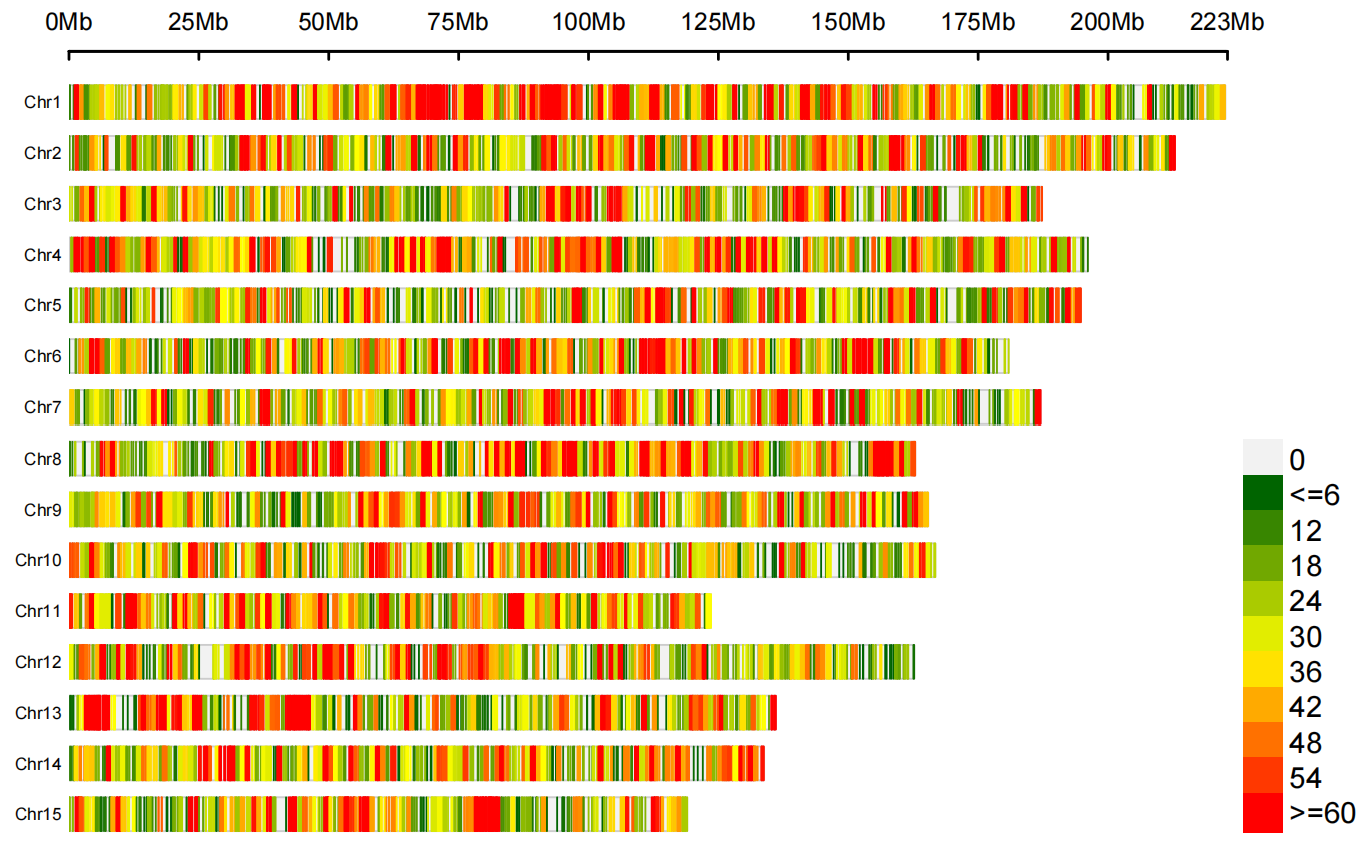


**Fig. S4** Distribution map of SNPs on 15 chromosomes graph

**Fig. S5** Graph for CV error in the range of *K* = 2 – 9 of 415 tea accessions

**A**

**
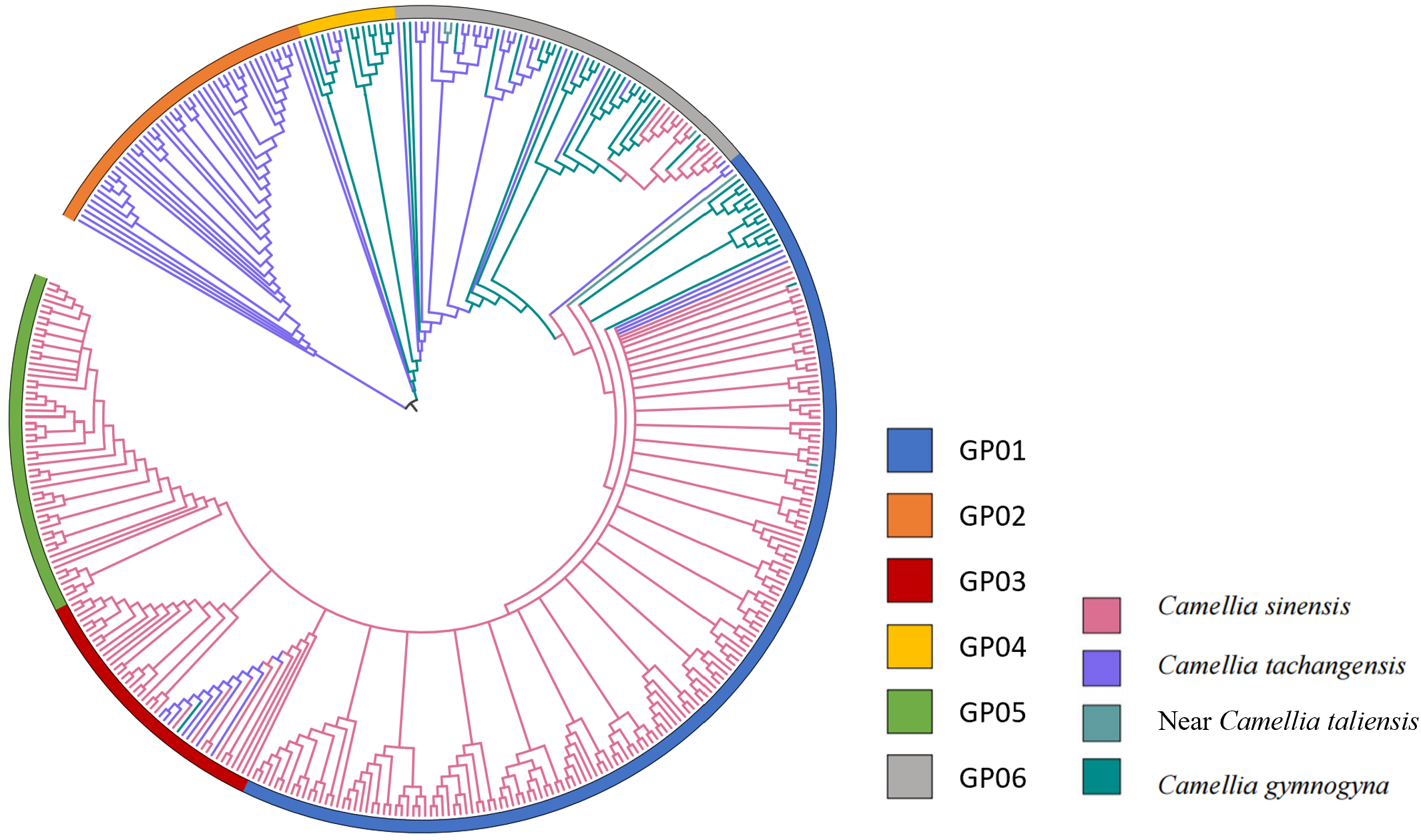
**

**B**


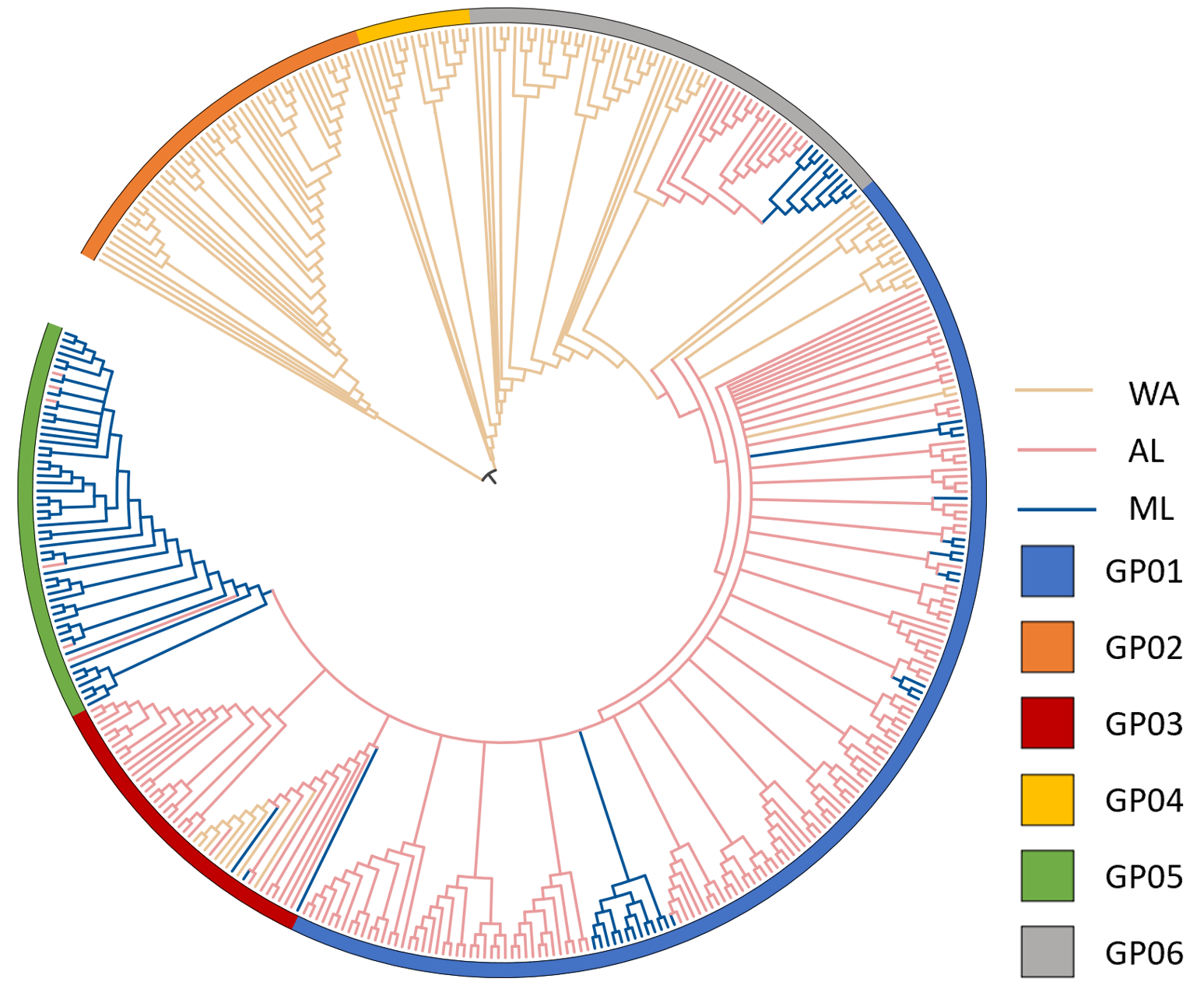


**Fig. S6** Cluster analysis using NJ trees. **A** NJ tree compared with classification of tea plants. *Camellia sinensis* (pink), *Camellia tachangensis* (purple), near *Camellia taliensis* (cyan) and *Camellia gymnogyna* (green). **B** NJ tree compared with cultivation status, wild type accessions (WA), ancient landraces (AL) and modern landraces (ML). The lines with different colors represent the species classification of tea plants and the cultivation status, and the squares with different colors nearby represent the populations we divided according to the results of ADMIXTURE software. GP01, GP02, GP03, GP04 and GP05 are pure populations and GP06 is admixture population

**A B**

**
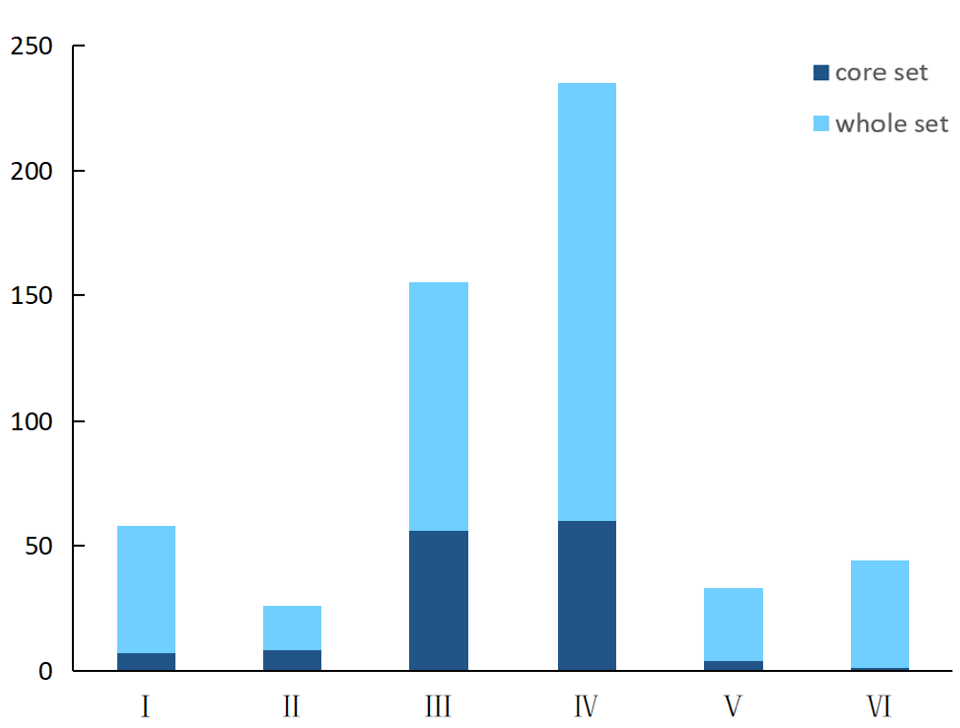
**
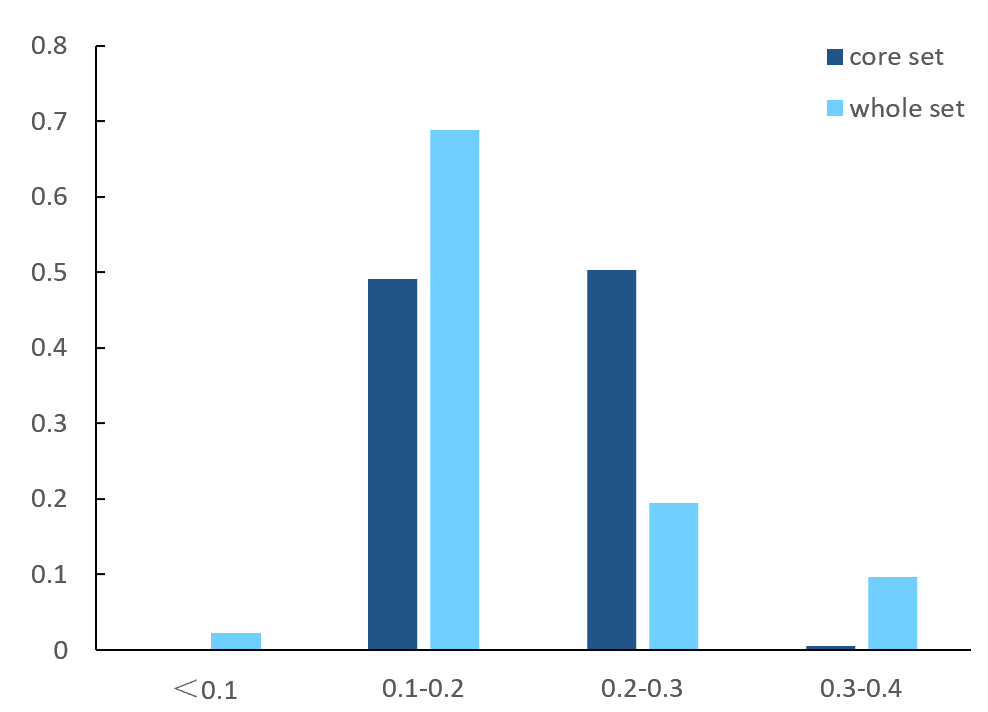


**C**

**
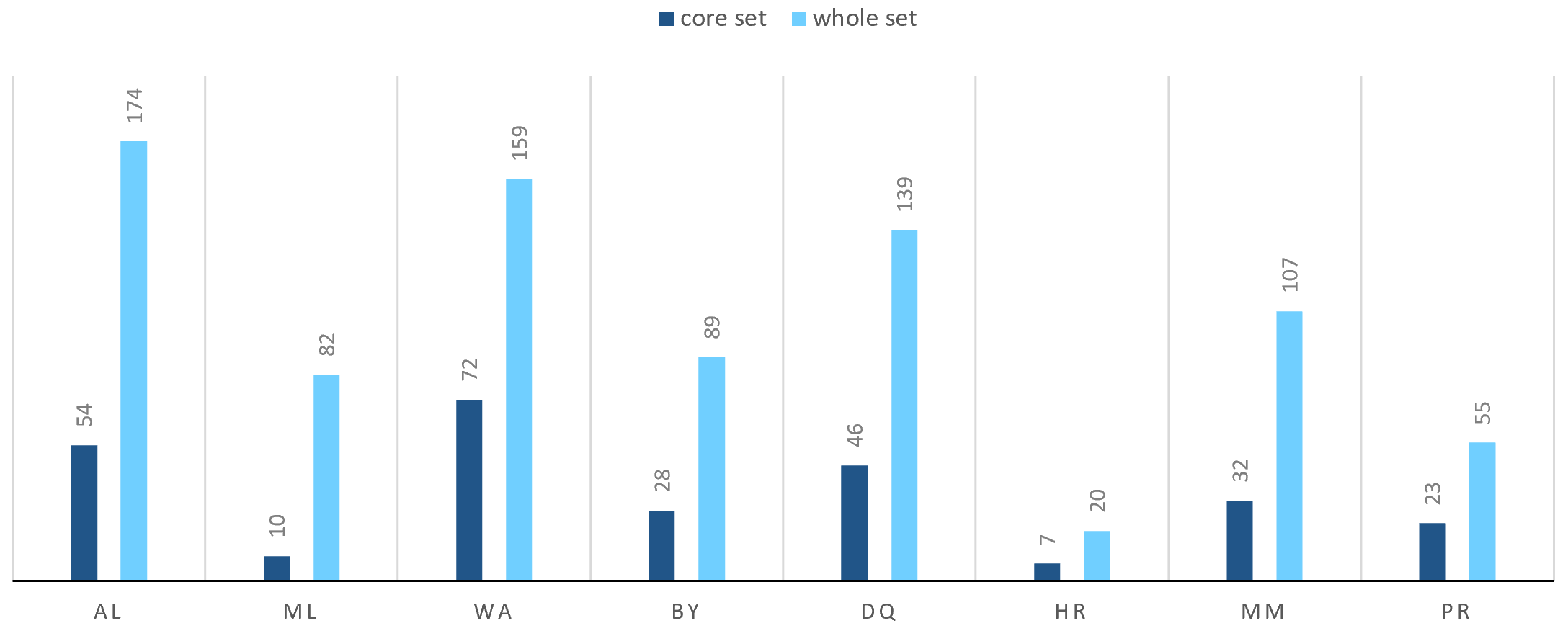
**

**Fig. S7** Summary of comparison information among core and whole sets. **A** Frequency distribution histogram of pairwise genetic distance of whole set and core set. **B** The histogram of the numbers of accessions of whole and core sets in six populations (Ⅰ to Ⅵ). **C** The histogram of the numbers of accessions of whole and core sets in cultivation status (wild type accessions (WA), ancient landraces (AL) and modern landraces (ML)) and indigenous communities (HR, DQ, BY, MM and PR indigenous communities)

**
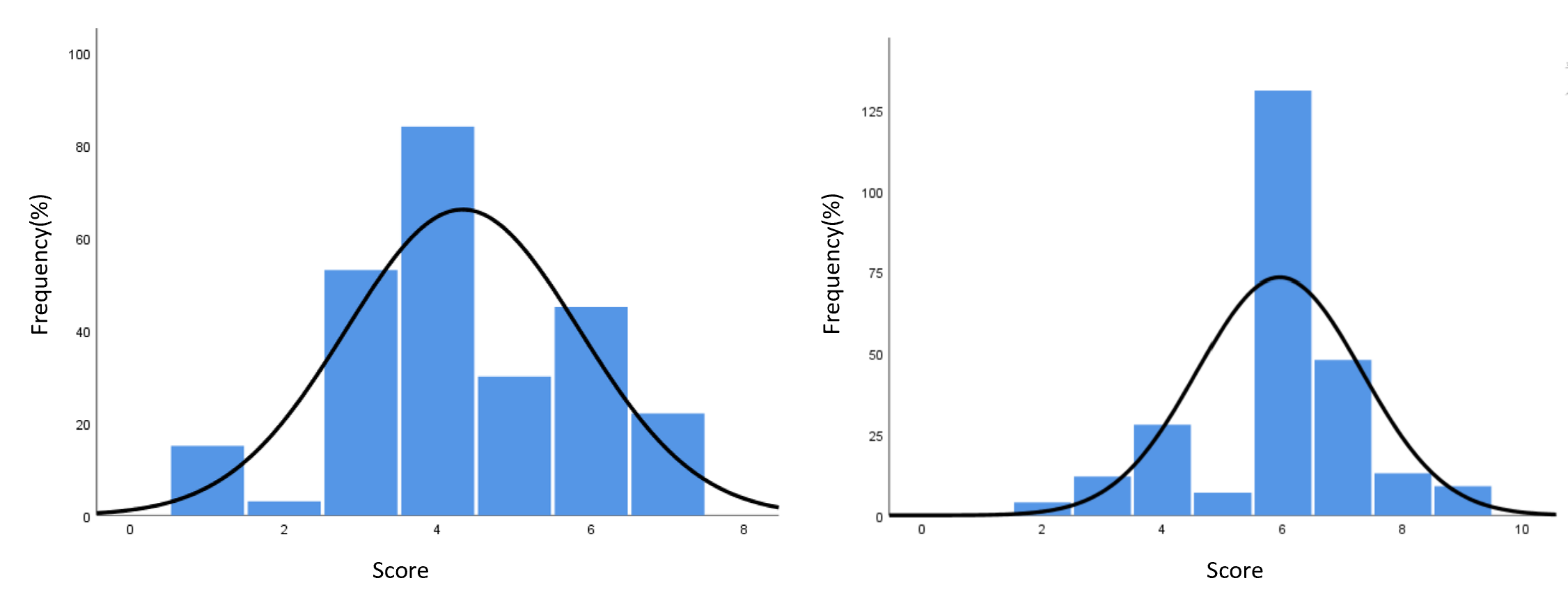
A B**

**Fig. S8** Phenotype frequency distribution of OTL and OtL. **A** Frequency distribution of OTL **B** Frequency distribution of OtL


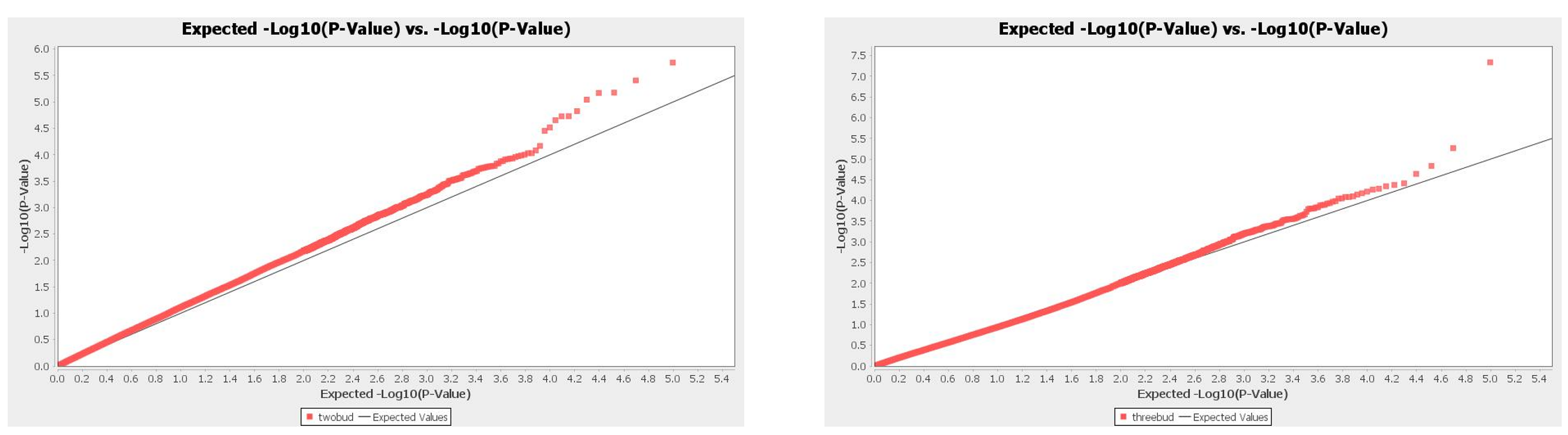
**A B**

**Fig. S9** GWAS analysis for OTL and OtL. **A** QQ plot for the OTL of tea plant by the optimal model of GLM. **B** QQ plot for the OtL of tea plant by the optimal model of MLM
